# Computer modeling defines the system driving a constant current crucial for homeostasis in the mammalian cochlea by integrating unique ion transports

**DOI:** 10.1038/s41540-017-0025-0

**Published:** 2017-08-25

**Authors:** Fumiaki Nin, Takamasa Yoshida, Shingo Murakami, Genki Ogata, Satoru Uetsuka, Samuel Choi, Katsumi Doi, Seishiro Sawamura, Hidenori Inohara, Shizuo Komune, Yoshihisa Kurachi, Hiroshi Hibino

**Affiliations:** 1 Department of Molecular Physiology, Niigata University School of Medicine, Niigata, Japan; 2 Center for Transdisciplinary Research, Niigata University, Niigata, Japan; 3 AMED-CREST, AMED, Niigata, Japan; 4 Department of Otorhinolaryngology, Graduate School of Medical Sciences, Kyushu University, Fukuoka, Japan; 5 Department of Physiology, School of Medicine, Toho University, Tokyo, Japan; 6 Department of Otorhinolaryngology–Head and Neck Surgery, Graduate School of Medicine, Osaka University, Suita, Japan; 7 Department of Electrical and Electronics Engineering, Niigata University, Niigata, Japan; 8 Department of Otolaryngology, Faculty of Medicine, Kindai University, Osakasayama, Japan; 9 Division of Otolaryngology–Head and Neck Surgery, Yuaikai Oda Hospital, Kashima, Japan; 10 Division of Molecular and Cellular Pharmacology, Department of Pharmacology, Graduate School of Medicine, Osaka University, Suita, Japan; 11 The Global Center for Medical Engineering and Informatics, Osaka University, Suita, Japan

**Keywords:** Physiology, Multicellular systems, Computer modelling

## Abstract

The cochlear lateral wall—an epithelial-like tissue comprising inner and outer layers—maintains +80 mV in endolymph. This endocochlear potential supports hearing and represents the sum of all membrane potentials across apical and basolateral surfaces of both layers. The apical surfaces are governed by K^+^ equilibrium potentials. Underlying extracellular and intracellular [K^+^] is likely controlled by the “circulation current,” which crosses the two layers and unidirectionally flows throughout the cochlea. This idea was conceptually reinforced by our computational model integrating ion channels and transporters; however, contribution of the outer layer’s basolateral surface remains unclear. Recent experiments showed that this basolateral surface transports K^+^ using Na^+^, K^+^-ATPases and an unusual characteristic of greater permeability to Na^+^ than to other ions. To determine whether and how these machineries are involved in the circulation current, we used an in silico approach. In our updated model, the outer layer’s basolateral surface was provided with only Na^+^, K^+^-ATPases, Na^+^ conductance, and leak conductance. Under normal conditions, the circulation current was assumed to consist of K^+^ and be driven predominantly by Na^+^, K^+^-ATPases. The model replicated the experimentally measured electrochemical properties in all compartments of the lateral wall, and endocochlear potential, under normal conditions and during blocking of Na^+^, K^+^-ATPases. Therefore, the circulation current across the outer layer’s basolateral surface depends primarily on the three ion transport mechanisms. During the blockage, the reduced circulation current partially consisted of transiently evoked Na^+^ flow via the two conductances. This work defines the comprehensive system driving the circulation current.

## Sensory neuroscience: let ion run through a cochlear labyrinth

In *in vivo* mammalian cochlea, ionic current constantly and unidirectionally flows—this unique “circulation current”, which contributes to high sensitivity of sensory cells transducing atomic scale acoustic vibrations to electrical signals, likely depends upon ion transports across a multiple-layered epithelial tissue. To determine how the circulation current is established, a team conducted by Hiroshi Hibino at Niigata University in Japan used a theoretical approach, because ionic currents are unmeasurable *in vivo*. A conceptual computational model they previously developed lacked involvement of an epithelial tissue membrane recently found to show unusual ion transport profile; integration and coupling of this element to other membrane transport machineries resulted in reproducing experimental measurements. This work defines the comprehensive system driving the circulation current, which remains uncertain for nearly 20 years, and helps us to understand the mechanism for hearing.

## Introduction

**Fig. 1:**
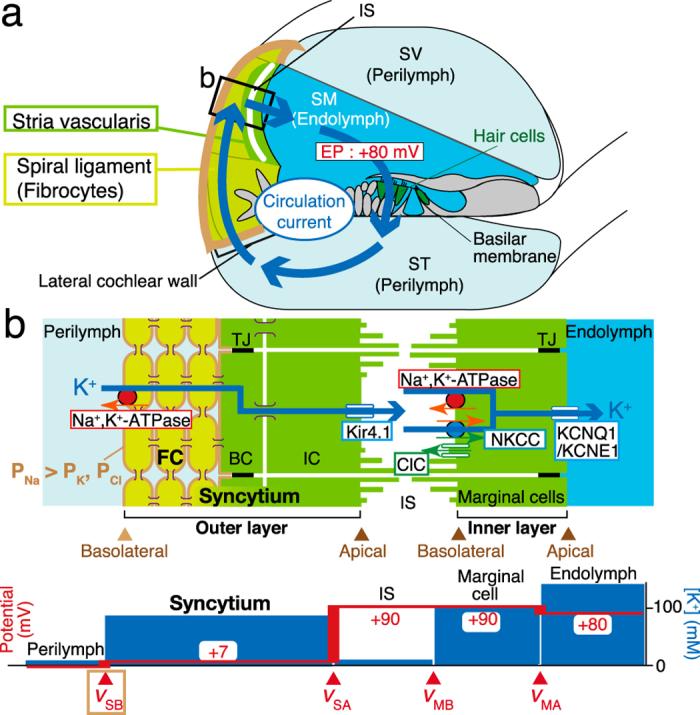
Morphological and electrochemical characteristics of the cochlea. **a** Structure of the cochlea. This organ is composed of three tubules: the scala vestibuli (SV), scala tympani (ST), and scala media (SM). The SV and ST contain a normal extracellular fluid, perilymph, whereas the SM is filled with the endolymph, of which [K^+^] and potential are 150 mM and +80 mV, respectively. This potential is referred to as an endocochlear potential (EP). The locations of the spiral ligament and stria vascularis, which constituent the lateral wall, are also illustrated. Note that the ligament is dominated by fibrocytes. The circulation current unidirectionally flows throughout the cochlea, as depicted. The cellular composition of the stria and ligament in the region indicated with a *black rectangle* is illustrated in the *upper panel* of **b**. **b** Structure of the lateral cochlear wall. In the lateral wall, fibrocytes (FC) of the ligament and the basal and intermediate cells of the stria (BC and IC, respectively) are all connected with gap junctions; therefore, they form a syncytium (*upper panel*). Because of the tight junctions (TJs) between the basal cells, fibrocytes and intermediate cells can be assumed to constitute the basolateral and apical surfaces in the syncytium, respectively. On the fibrocyte membrane, permeability of Na^+^ is larger than permeability of K^+^ and Cl^−^ (P_Na_ > P_K_, P_Cl_).^30^ Between syncytial and marginal-cell layers in the stria lies the intrastrial space (IS), an extracellular compartment whose width is 15 nm. Channels and transporters shown in *the upper panel* likely perform pivotal functions in maintaining the circulation current and EP. The syncytium and the layer of the other strial cell type, marginal cells, are comparable to the outer and inner layers, respectively (see Abstract and Introduction). The *lower panel* shows electrochemical properties of each compartment of the lateral wall; *v*_SB_, *v*_SA_, *v*_MB_, and *v*_MA_ correspond to the membrane potentials across the basolateral and apical surfaces of the syncytial and marginal-cell layers

The electrochemical balance between intracellular and extracellular compartments is crucial for proper activity of all the tissues and organs. The cochlea of the mammalian inner ear, a peripheral organ of hearing, requires specific ionic and potential environments to effectively transduce the mechanical energy of sounds into electrical signals. This organ comprises three chambers: the scala media, scala tympani, and scala vestibuli (Fig. 1a). The scala tympani and scala vestibuli contain perilymph, which maintains ionic composition of a regular extracellular fluid. On the other hand, the scala media is filled with an unordinary extracellular solution that always contains high [K^+^], 150 mM, and a high potential of +80 mV relative to perilymph, called endocochlear potential (EP).^1, 2^ A sensory epithelial layer of hair cells exposes the apical surface to the endolymph of the scala media and immerses the basolateral surface in the perilymph of the scala tympani. Sound input activates mechanoelectrical transduction (MET) channels on the stereocilia protruding from the apical site of hair cells. This process introduces endolymphatic K^+^ into the cells, depolarizing them.^3^ The highly positive EP greatly sensitizes hair cells by accelerating the K^+^ entry. K^+^, after exciting hair cells, exits to perilymph via K^+^ channels on the basolateral surface along the electrochemical gradient, allowing the cells to recover their ionic milieu back to the resting condition without consuming energy.^4–9^ Accordingly, the endolymphatic high [K^+^] as well as EP are essential for hearing. These properties are likely to be maintained by the lateral cochlear wall, an epithelial-like tissue composed functionally of outer and inner layers.^10–12^ EP represents the sum of all the membrane potentials on the apical and basolateral surfaces of the two layers^11, 13–15^ (Fig. 1b). In vivo electrophysiological experiments have revealed that the apical surface in each layer is governed by K^+^ equilibrium potential (*E*_K_), which depends on intracellular and extracellular [K^+^] across the membrane (Fig. 1b).^13–15^ An outline of the regulatory mechanisms underlying the [K^+^] properties was shown by our earlier computational model based on experimental observations as follows.^16, 17^ First, on each basolateral and apical surface in the outer and inner layers of the lateral wall, ion channels and transporters are engaged in an interplay and unidirectionally transport K^+^ under normal conditions. Second, these four types of unidirectional K^+^ transport that take place in the lateral wall and the transcellular K^+^ flow of the hair-cell layer are linked, circulating K^+^ continuously between endolymph and perilymph (Fig. 1a). Third, this circulation current, namely K^+^ recycling,^11, 18, 19^ controls the lateral wall’s intracellular and extracellular [K^+^] properties underlying the EP. The circulation current has been recorded in vivo as the potential gradient stemming from ionic fluxes using a probe placed in perilymph.^6^ This current likely contributes to the maintenance of not only EP but endolymphatic high [K^+^].^12, 18–21^ Similar ionic circulation seems to occur in retinal photoreceptors and intestinal epithelia and may be involved in their electrochemical homeostasis.^22, 23^

Among the four membrane domains of the lateral cochlear wall, the apical and basolateral surfaces of the inner layer and the apical surface of the outer layer have been extensively analyzed by different approaches^8, 12, 17, 21^; the relevance of the channels and transporters to the circulation current has also been theoretically described in the aforementioned computational model.^16^ In this model, the unidirectional K^+^ transport across the outer layer’s basolateral surface, which faces perilymph, was simply designed with histochemically identified Na^+^,K^+^-ATPases and Na^+^,K^+^,2Cl^−^-cotransporter (NKCC)^24–27^ although physiological significance of these two K^+^ uptake transporters had remained elusive. In this context, the modeling for the circulation current has not yet been completed. Recent experimental endeavors have revealed that in the outer layer’s basolateral surface, Na^+^,K^+^-ATPases but not NKCC are likely to transport K^+^ in vivo.^28, 29^ Furthermore, this surface is more permeable to Na^+^ than to K^+^ and Cl^−^, and therefore it shows positive resting membrane potential (RMP) of +5 to +12 mV with reference to perilymph.^28, 30^ This permeability profile is relatively unique because in the majority of cell types, K^+^ or Cl^−^ permeability dominates the membrane in the resting state.^31–34^ In the present study, we theoretically determine whether and how the ion transport mechanisms on the outer layer’s basolateral surface contribute to the circulation current essential for hearing.

## Model development

### Formation of EP

Morphological characteristics of the lateral cochlear wall are depicted in Fig. 1b. The lateral wall consists of two tissue components: the stria vascularis and spiral ligament. The stria vascularis contains marginal, intermediate, and basal cells, whereas the spiral ligament comprises fibrocytes. Marginal cells constitute a single epithelial layer that is apically exposed to endolymph. Intermediate cells, basal cells, and fibrocytes are connected via gap junctions, so that these three cell types constitute an electrochemical syncytium.^18, 19, 35^ This syncytium serves as an epithelial-like layer; the basolateral surface is primarily composed of the fibrocytes’ membranes bathed in perilymph, and the apical surface is dominated by intermediate cells’ membranes facing the basolateral membranes of marginal cells. The marginal-cell and syncytial layers correspond to the aforementioned inner and outer layers, respectively; in the text below, the first two terms will be used. In these two layers, tight junctions of marginal cells and basal cells act as physical and electrical barriers, as observed in common epithelial tissues.^23, 36–39^ Because intermediate cells’ membranes and basolateral membranes of marginal cells are highly interdigitated, the extracellular space between the two layers is narrow: ~15 nm.^40^ This so-called intrastrial space (IS) has a low [K^+^] of 5 mM and a highly positive potential similar to EP.^13, 15, 41^

In accordance with some studies,^11, 13–15^ EP and the potential of the IS (ISP) are defined as follows:

1 $${\rm{EP}} = {v_{{\rm{SB}}}} - {v_{{\rm{SA}}}} + {v_{{\rm{MB}}}} - {v_{{\rm{MA}}}}$$

and

2 $${\rm{ISP}} = {v_{{\rm{SB}}}} - {v_{{\rm{SA}}}},$$

where *v*_SB_ and *v*_SA_ are the membrane potentials across the basolateral and apical surfaces of the outer syncytial layer, respectively, and *v*_MB_ and *v*_MA_ are those across the basolateral and apical surfaces of the inner marginal-cell layer, respectively (Fig. 1b). Each membrane potential is measured with reference to the neighboring extracellular fluid. Under physiological conditions, the voltage difference across the marginal-cell layer (*v*_MB_ − *v*_MA_) is small: <10 mV^13, 15^; therefore, EP is governed primarily by ISP.^14^

### General description of the computational model

**Fig. 2:**
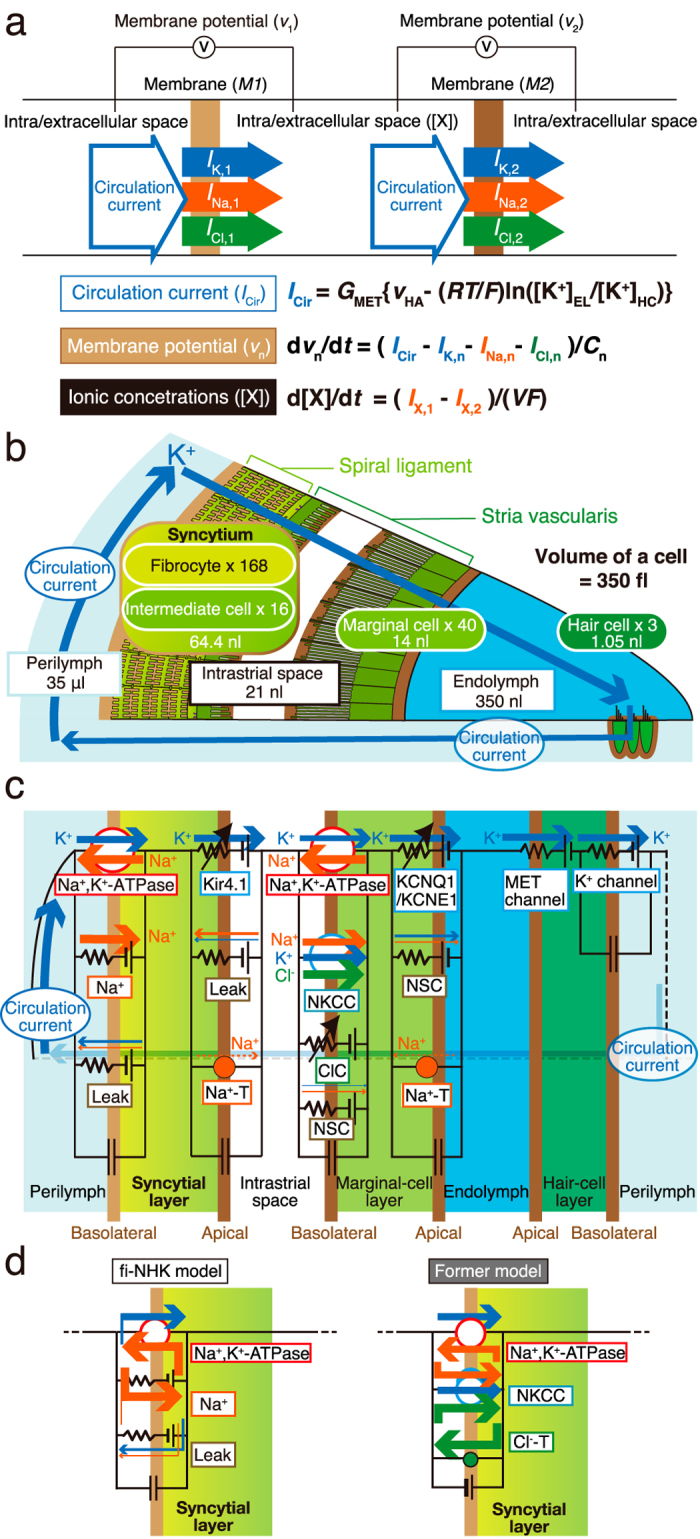
Principles and key elements of the fi-NHK model. **a** Membrane currents and spatial electrochemical properties defined in the fi-NHK model. The schematic shows a general model of current flows through a single layer or layer–layer interface. The circulation current (*I*_Cir_), which passes through all the membranes and spaces, corresponds to the sum of ionic flows through three hair cells; therefore, it depends on the conductance of mechanoelectrical transduction channels (*G*_MET_) and the potential difference between endolymph and the hair-cell layer (*v*_HA_; *first equation*). Also illustrated are currents carried by three ion species, K^+^, Na^+^, and Cl^−^, which flow through channels and transporters across membrane 1 (*M1*) into the neighboring space (*I*_K,1_, *I*_Na,1_, and *I*_Cl,1_, respectively) and leave from this space across membrane 2 (*M2*) (*I*_K,2_, *I*_Na,2_, and *I*_Cl,2_, respectively). In this situation, a change in the membrane potential across *M1* (*v*_1_) depends on the difference between *I*_Cir_ and the sum of *I*_K,1_, *I*_Na,1_, and *I*_Cl,1_. This is also the case for the membrane potential across *M2*, as summarized in the *second equation* where *n* indicates the ID number of a membrane. A change in each ion concentration in the space is calculated by subtraction of the ionic flow across *M2* from that across *M1* as denoted in the *third equation*. In this equation, each ionic species is represented by X. [K^+^]_EL_ and [K^+^]_HC_ are K^+^ concentrations in endolymph and the hair cell, *C*_n_ is the capacitance of membrane n, *V* is the volume of the intracellular or extracellular space, and *F*, *R*, and *T* are the Faraday constant, Gas constant, and absolute temperature, respectively. For details, see also “General description of the computational model.” **b** Components of the model. The number of cells of each type and the volume of each tissue are shown with the volume of perilymph, of the intrastrial space, and of endolymph. **c**, **d** All the ionic flows in the fi-NHK model. In **c**, all the intracellular compartments and membrane domains constructed in the model are also illustrated and highlighted in *graded green* and *brown*, respectively. In the model, syncytial, marginal-cell, and hair-cell layers are assembled in a series. Ion conductance and transporters form a closed-loop circuit which serves as the route for the circulation current (**c**). In the former NHK model (*right panel* in **d**), the syncytial basolateral surface was provided with Na^+^,K^+^-ATPases, Na^+^,K^+^,2Cl^−^-cotransporter (NKCC), and Cl^−^ transporter (Cl^−^-T). In the updated (fi-NHK) model (**c** and *left panel* in **d**), this surface is assumed to harbor only Na^+^,K^+^-ATPases, Na^+^ conductance, and leak conductance and to locally recycle Na^+^ among these three machineries. NKCC, which has been immunohistochemically detected in fibrocytes, appears to only minimally contribute to K^+^ transport,^29^ and therefore was not included in the fi-NHK model (also see **c**). *Na*^+^ Na^+^ conductance, *Leak* leak conductance, *NSC* nonselective cation conductance, *Na*^+^*-T* Na^+^ transporter, *MET* mechanoelectrical transduction, *ClC* ClC/K Cl^−^ channels

The computational model constructed and used in this study is an updated version of the Nin–Hibino–Kurachi (NHK) model described in our earlier work,^16^ and therefore principles of the two are basically the same. The present model, which was constructed using MATLAB 2016b (Mathworks, Natick, US), was designed to simulate electrochemical phenomena of the cochlea in 10-µm slices, a distance corresponding to the width of one row of hair cells and lateral-wall cells. All the parameters used in the model are shown in Supplementary Table 1 (see also the section “Parameter settings” mentioned below). Besides aforementioned Eqs. (1, 2), pivotal equations defining the model are described below and in Fig. 2a; more detailed formulations are shown in Supplementary Information. Briefly, the amplitudes and characteristics of EP, ISP, and the circulation current depended upon functionality of ion channels, ion transporters, and ion permeability on the membrane as well as morphological profiles of the cochlea, which includes three layers; outer syncytial, inner marginal-cell, and hair-cell layers, and three extracellular spaces; the IS, perilymph in the scala tympani and scala vestibuli, and endolymph in the scala media (Fig. 2b, c). To reproduce the circulation current flowing throughout the cochlea,^4, 6^ a large closed-loop circuit was constructed by connecting the ion transport mechanisms in six membrane domains (i.e., six surfaces) in a series, as illustrated in Fig. 2c. In this arrangement, the circulation current (*I*_Cir_) is always comparable to the summed current through MET channels of the hair-cell layer (MET current; *I*_MET_). Because the amplitude of *I*_MET_ is proportional to the potential difference across the apical surface of the hair-cell layer; in other words, the value obtained by subtraction of EP from the membrane potential across the basolateral surface of the hair-cell layer,^4^ the value of *I*_*Cir*_ (i.e., *I*_MET_) depends on that of EP as follows^16^ (Fig. 2a):

3 $${v_{{\rm{HA}}}} = - {\rm{EP}} + {v_{{\rm{HB}}}}$$

4 $${I_{{\rm{MET}}}} = {G_{{\rm{MET}}}}\left( {{v_{{\rm{HA}}}} - \frac{{RT}}{F} \ {\rm{ln}}\left( {\frac{{{{\left[ {{{\rm{K}}^ + }} \right]}_{{\rm{EL}}}}}}{{{{\left[ {{{\rm{K}}^ + }} \right]}_{{\rm{HC}}}}}}} \right)} \right),$$

where [K^+^]_EL_ and [K^+^]_HC_ are [K^+^] in endolymph and the hair cell, respectively, *G*_MET_ denotes the conductance of MET channels, *v*_HA_ and *v*_HB_ are the potentials of the hair-cell apical and basolateral surfaces, respectively, *R* is the gas constant, *T* represents temperature, and *F* is the Faraday constant. The MET current exists even without acoustic stimuli (~1 nA/hair cell)^42, 43^; therefore, *I*_Cir_ flows throughout the cochlea under physiological conditions.

The definitions and relations among membrane potentials and extracellular or intracellular ion concentrations are as follows (Fig. 2a). The change in the membrane potential of the apical and basolateral surface of each layer obeys the difference between *I*_Cir_ that flows into the membrane and the sum of currents that move through the channels and transporters on the same membrane (*I*_M_):

5 $$\frac{{{\rm{d}}v}}{{{\rm{d}}t}} = \frac{1}{C} \frac{{{\rm{d}}Q}}{{{\rm{d}}t}} = \frac{{{I_{{\rm{Cir}}}} - {I_{\rm{M}}}}}{C} = \frac{{{I_{{\rm{Cir}}}} - {I_{\rm{K}}} - {I_{{\rm{Na}}}} - {I_{{\rm{Cl}}}}}}{C},$$

where *v* is the membrane potential, *Q* is the electric charge accumulated on the membrane, *C* denotes capacitance of the membrane, and *I*_K_, *I*_Na_, and *I*_Cl_ represent K^+^, Na^+^, and Cl^−^ current fractions, respectively, constituting *I*_M_. Note that in the lateral wall, *I*_Cir_ also stems from the sum of all the currents through the channels and transporters. Furthermore, the ion concentrations in each extracellular and intracellular compartment are regulated by ionic currents through the membranes:

6 $$\frac{{{\rm{d}}[\text{X}]}}{{{\rm{d}}t}} = \frac{{{I_{{\rm{X}},{\rm{In}}}} - {I_{{\rm{X}},{\rm{Out}}}}}}{{V F}},$$

where [X] is the concentration of ion X, *I*_X,In_ represents the inward current of X, *I*_X,Out_ is the outward current of X, and *V* denotes the volume of the compartment. *I*_X,In_ and *I*_X,Out_ are constituents of *I*_M_ and *I*_Cir_. Thus, in the steady state, *I*_Cir_ is equivalent to *I*_M_ for all membranes, and *I*_X,In_ is the same as *I*_X,Out_ for all the compartments, providing constant values for all ionic concentrations (d[X]*/*d*t* = 0), all membrane potentials (d*v/*d*t* = 0), and both EP and ISP, which derive from the membrane potentials (Eqs. (1, 2)). If the channels or transporters are modulated by certain perturbations, a difference between *I*_M_ and *I*_Cir_ emerges; consequently, the membrane potentials, ionic concentrations, and EP change.

### Ion channels and transporters

Detailed equations for each type of ion channels and transporters incorporated into our model are shown in Supplementary Information; the principles are described below.

The current through ion channels in the model obeys Ohm’s law:

7 $${I_{{\rm{Channel}}}} = {G_{{\rm{Channel}}}} \left( {v - {E_{\rm{X}}}} \right),$$

where *I*_Channel_ is the current through a channel permeable to ion X, *G*_Channel_ is conductance of the channel, *v* is the membrane potential in which the channel is located, and *E*_X_ is the equilibrium potential of ion X.

The function of ion transporters is regulated by different sets of parameters, which vary among the transporter types. In our model, the net current through a transporter (*I*_Transporter_) is defined as follows:

8 $${I_{{\rm{Transporter}}}} = f\left( {v,{{\left[ {{{\rm{K}}^ + }} \right]}_{{\rm{in}}}},{{\left[ {{{\rm{K}}^ + }} \right]}_{{\rm{out}}}},{{\left[ {{\rm{N}}{{\rm{a}}^ + }} \right]}_{{\rm{in}}}},{{\left[ {{\rm{N}}{{\rm{a}}^ + }} \right]}_{{\rm{out}}}},{{\left[ {{\rm{C}}{{\rm{l}}^ - }} \right]}_{{\rm{in}}}},{{\left[ {{\rm{C}}{{\rm{l}}^ - }} \right]}_{{\rm{out}}}}} \right),$$

where *v* is the membrane potential, and [K^+^]_in_, and [K^+^]_out_, [Na^+^]_in_, [Na^+^]_out_, [Cl^−^]_in_, and [Cl^−^]_out_ are respective ionic concentrations inside and outside the cell.

### Parameter settings

All the parameters used in the model such as the conductances and activities of channels and transporters, membrane capacitances, the number of cells of each type, and the volume of each intra/extracellular space are based on the experimental observations and are listed in Supplementary Table 1. All the initial values of the spatial ion concentrations and membrane potentials applied to begin the simulations were also determined on the basis of the measured data and are shown in Supplementary Table 2. Procedures for setting several crucial parameters are described below.

### Morphometry of the tissues in the lateral cochlear wall

In the updated version of the NHK model, we made the volumes equal for all the cell types in the lateral wall and for hair cells (Supplementary Table 1), as we proposed in the former model.^16^ Therefore, the number of cells constituting the stria vascularis or spiral ligament correlates with the volume of the respective tissue, except for the extracellular space. To determine the number of fibrocytes for the simulation, we histologically analyzed a cochlear section of guinea pigs as follows. All the animal experiments, which were designed in accordance with the Japanese Animal Protection and Management Law, were carried out in compliance with the protocol that was reviewed by the Institutional Animal Care Committee and the President of Niigata University (Permission Number: #26 Niigata Univ. Res. 96–1). Guinea pigs were housed at the animal facility and kept in a 12-h light/12-h dark cycle. Food and water were provided ad libitum. All animal handling and reporting comply with the ARRIVE guidelines.^44^ Cochlear samples were prepared as described previously.^45^ Male Hartley guinea pigs (200–400 g, 3–5 weeks of age; SLC Inc., Hamamatsu, Japan) were intraperitoneally administrated with pentobarbital sodium (150 mg/kg; Somnopentyl; Kyoritsu Seiyaku, Tokyo, Japan). The depth of anesthesia was confirmed by the absence of a response to a toe pinch and by the corneal reflex. After that, the animals were perfused through their left ventricle with 4% paraformaldehyde in 0.1 M sodium phosphate, pH 7.4. The cochlea samples isolated from the temporal bone were prepared as cryothin sections (5 µm) by Kawamoto’s film method.^46^ These slices were stained with hematoxylin and eosin and examined under a light microscope (FSX-100, Olympus, Tokyo, Japan).

First, an image of the stria vascularis and spiral ligament in the second turn of the cochlear cross-section was captured at low magnification (×75) using a microscope. In this image, the edge of each tissue was traced by means of the “Measurement and ROI tool” in the cellSens software (Olympus) to measure the area (Supplementary Fig. 1a). The total regions of the stria and ligament had the area of 12,107.1 ± 798.4 and 74,353.8 ± 4709.5 μm^2^ (*n* = 3), respectively. Next, in a high-magnification image (×1000; Supplementary Fig. 1b, c), the proportion of cell bodies including a nucleus in each tissue was determined by means of the “Manual HSV threshold tool” in the software (the stria: 91.6% ± 4.5%, the spiral ligament: 42.3% ± 1.6%; n = 3). By calculating these percentages and the area, we found that the ratio of the total cellular region in the stria to that in the ligament was approximately 1:3. This ratio is comparable to the ratio of tissue volumes excluding the extracellular space in the 10-µm slice of the cochlea and therefore corresponds to the ratio of the cell numbers. In our previous study,^16^ the number of strial cells composed primarily of marginal and intermediate cells (MCs and ICs, respectively) was estimated to be 56 per slice (*N*_MC = _16 and *N*_IC_ = 40; Fig. 2b). Thus, in the present study, the number of fibrocytes, which dominate cellular components of the ligament, was set to 168 (Fig. 2b and Supplementary Table 1).

### Conductances and activities of the ion transport apparatus

All the channels and transporters integrated into the model and all the ionic currents carried by this ion transport apparatus are illustrated in Fig. 2c. Major revisions of the previous model are as follows (Fig. 2d): In the present model, we omitted histochemically detected NKCC from the syncytial basolateral surface and made sure that in this membrane domain, the K^+^ transport, which likely mediates the circulation current, was governed primarily by Na^+^,K^+^-ATPases (see Introduction). In addition, our in vivo assays indicated that the syncytial basolateral surface has a positive RMP of +5 to +12 mV because it is more permeable to Na^+^ than to K^+^ and Cl^−^.^30^ In the experiments, perilymphatic perfusion with a low-Cl^−^ solution moderately hyperpolarized *v*_SB_. This perturbation was expected to cause depolarization if the membrane harbored significant Cl^−^ conductance; therefore, its manifestation is unlikely. Existence of significant K^+^ conductance could also be ruled out for two reasons. First, the perilymphatic perfusion with various K^+^ channel blockers had only moderate effects on EP.^47, 48^ Second, a reduction in [K^+^] in the syncytial layer ([K^+^]_SY_) by a pharmacological intervention led to hyperpolarization of *v*_SB_.^28, 29^ These recently identified profiles were simply reproduced in the updated model with a few processes as follows (Fig. 2c, d). We incorporated Na^+^ and leak conductances but none of the other conductances into the basolateral surface of the syncytial layer to represent its permeability profile mentioned above. Moreover, the value of Na^+^ conductance was set to significantly exceed that of leak conductance. No Cl^−^ transport was included in the syncytial basolateral surface.

Accordingly, the total current through the basolateral surface of the syncytial layer (*I*_SB_) is described by the formula

9 $${I_{{\rm{SB}}}} = {I_{{\rm{NaKATP,SB}}}} + {I_{{\rm{NaConductance,SB}}}} + {I_{{\rm{Leak,SB}}}},$$

where *I*_NaKATP,SB_ is the net ionic flow through Na^+^,K^+^-ATPases, and *I*_NaConductance,SB_ and *I*_Leak,SB_ are the currents through Na^+^ conductance and leak conductance, respectively. The activity of Na^+^,K^+^-ATPases and values of Na^+^ conductance and leak conductance were determined as follows. Under physiological conditions, the membrane potential of the basolateral surface of the syncytial layer and ionic concentrations inside this layer are constant. Therefore, a net current through the basolateral surface of the syncytial layer corresponds to *I*_Cir_, and total K^+^ inflow and outflow are equal in this layer. In this context, we proposed that Na^+^ excreted by Na^+^,K^+^-ATPases and leak conductance immediately flows into the syncytial layer through Na^+^ conductances, thus representing local Na^+^ recycling among the three machineries (Fig. 2d). These relations are described by Eqs. (10–12):

10 $${I_{{\rm{NaKATP}},{\rm{SB}}}} + {I_{{\rm{NaConductance}},{\rm{SB}}}} + {I_{{\rm{Leak}},{\rm{SB}}}} = - {I_{{\rm{Cir}}}},$$

11 $${I_{{\rm{K}},{\rm{NaKATP}},{\rm{SB}}}} + {I_{{\rm{K}},{\rm{Leak}},{\rm{SB}}}} = - {I_{{\rm{K}},{\rm{Net}},{\rm{SA}}}},$$

12 $${I_{{\rm{Na}},{\rm{NaKATP}},{\rm{SB}}}} + {I_{{\rm{NaConductance}},{\rm{SB}}}} + {I_{{\rm{Na}},{\rm{Leak}},{\rm{SB}}}} = 0,$$

where *I*_K,NaKATP,SB_ and *I*_Na,NaKATP,SB_ are the K^+^ and Na^+^ flows through Na^+^,K^+^-ATPases, *I*_K,Leak,SB_ and *I*_Na,Leak,SB_ are the K^+^ and Na^+^ current components through the leak conductance on the basolateral surface of the syncytial layer, and *I*_K,Net,SA_ is the net K^+^ current through the apical surface (see Supplementary Information for details). In a steady state, *I*_K,Net,SA_ is equivalent to *I*_Cir_. The relations among Eqs. (10–12) indicate that Na^+^ conductance is constitutively active because its inward current correlates with Na^+^ outflow through Na^+^,K^+^-ATPases that continuously work and contribute to the circulation current. We solved Eqs. (10–12) simultaneously using the ionic concentrations and potentials measured in various extracellular or intracellular spaces and membrane domains in vivo under physiological conditions, respectively (Supplementary Table 2), and obtained values of Na^+^ and leak conductance and activity of the ATPases (Supplementary Table 1). Taken together, the arrangements on the syncytial basolateral surface mirror the assumption that the membrane potential and circulation current on this membrane rely on Na^+^ and leak conductances and Na^+^,K^+^-ATPases.

Other parameters of ion channels and transporters in the model were also based on experimental data, and are listed in Supplementary Table 1.

### The blocking rate of syncytial Na^+^,K^+^-ATPases during perilymphatic perfusion with ouabain

Perilymphatic perfusion with ouabain causes dysfunction of Na^+^,K^+^-ATPases on the basolateral surface of the syncytial layer. Because the extent of blockage of an ATPase has not been measured experimentally, we used estimated blocking rates to simulate the effect of ouabain. The blocking rate of the fibrocyte ATPases (*κ*_Ouabain,FC_) was determined as follows. First, in the model, we inhibited the Na^+^,K^+^-ATPases to different degrees (*κ*_Ouabain,FC_: 0.3 to 0.6) (Supplementary Fig. 2a). Next, we compared the simulated values of EP, *v*_SB_, [K^+^]_SY_, ISP, and [K^+^]_IS_ at 40 min after the onset of the inhibition with the stable values that were measured at 40 min after applying 10 μM ouabain to perilymph in the present study (see “Results”). This comparison is displayed in Supplementary Fig. 2a. When *κ*_Ouabain,FC_ was set to 0.46, all the simulated data reached values within the ranges of the experimental measurements as summarized in Table 1. Accordingly, we used this value (0.46) to reproduce the effects of ouabain perfusion (Supplementary Table 1).

**Table 1:** Comparison of simulated results and experimental measurements

Potential or [K^+^]	Simulation		Experiment		
	Normal^a^	Na^+^,K^+^-ATPase block^b^	Control^c^	Ouabain^d^	*p* value
EP	+72.7 mV	+9.6 mV	+83.2 ± 3.6 mV (*n* = 4)	+5.8 ± 6.2 mV (*n* = 4)	0.0002
*v*_SB_	+9.6 mV	−3.0 mV	+9.9 ± 1.2 mV (*n* = 3)	−6.2 ± 2.6 mV (*n* = 3)	0.0170
[K^+^]_SY_	98.3 mM	10.4 mM	101.7 ± 15.1 mM (*n* = 3)	14.3 ± 1.5 mM (*n* = 3)	0.0082
ISP	+81.2 mV	+15.1 mV	+68.7 ± 4.6 mV (*n* = 4)	+10.6 ± 4.4 mV (*n* = 4)	0.0004
[K^+^]_IS_	6.1 mM	4.7 mM	8.6 ± 2.5 mM (*n* = 4)	4.2 ± 1.4 mM (*n* = 4)	0.0051

*EP* endocochlear potential, *v*_*SB*_ the membrane potential across the basolateral surface of the syncytial layer, [*K*^+^]_*SY*_: [*K*^+^] inside the syncytial layer, *ISP* intrastrial potential, [*K*^+^]_*IS*_: [*K*^+^] in the intrastrial space

^a^ The steady-state values developed during 600 s after the onset of the calculation (Supplementary Table 2)

^b^ Values determined by the simulation at 40 min after the onset of Na^+^,K^+^-ATPase blockade

^c^ Values collected from the experimental data at 5 min before the onset of ouabain perfusion

^d^ Values obtained at 40 min after the onset of ouabain perfusion

## Results

### Validation of the updated model by experimental results

Our previous model could not reproduce changes in *v*_SB_ and [K^+^]_SY_ because of a lack of ion transport machineries on the basolateral surface of the syncytial layer^16^ (Fig. 2d). *v*_SB_ was hyperpolarized whereas [K^+^]_SY_ and EP were reduced during the perfusion of ouabain, an inhibitor of Na^+^,K^+^-ATPases, into a perilymphatic space: the scala tympani.^28, 29^ In the present study, we attempted to test whether these experimental observations could be replicated by our updated “fibrocyte-integrating Nin-Hibino-Kurachi (fi-NHK) model” for its overall evaluation. In preliminary experiments, we succeeded in reducing the size of fenestrae (<100 μm in diameter) for insertion of electrophysiological microelectrodes into the cochlea, as compared to earlier works (fenestrae ≥ 200 μm in diameter).^29, 30^ This improvement must decrease the damage to the cells constituting the lateral wall (see Discussion). Accordingly, we first repeated the in vivo experiments described above.

**Fig. 3:**
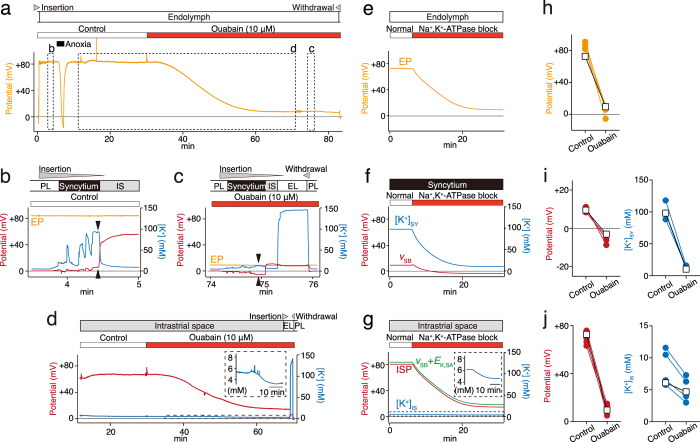
Experimental measurements and simulation of the potential and [K^+^] in the cochlea. **a**–**d** In vivo assays by electrophysiological methods. While EP was continuously recorded with a single-barreled microelectrode placed in the scala media, control artificial perilymph or a solution containing ouabain (10 µM) was perfused into the scala tympani in the period indicated by the *bars* above the trace (**a**). Administration of the guinea pig with anoxia induced a reduction in EP to a negative value as reported elsewhere.^55^ Double-barreled microelectrodes were transiently inserted from the perilymph of the scala tympani (*PL*) toward the endolymph (*EL*) of the scala media twice during the periods marked by *dashed boxes* in **a**. During each insertion, the potential (*red*) and [K^+^] (*blue*) were recorded (**b**–**d**). Note that recordings of **b**, **d** were performed sequentially during the first insertion. The traces of EP (*orange*) are overlaid in **b**, **c**. In these panels, the compartment immediately prior to the intrastrial space (IS) represents the profile within the syncytial layer (*arrowheads*). In **d**, the double-barreled microelectrode was located in the IS for approximately an hour and then advanced to EL. The [K^+^]_IS_ dynamics marked by a *dashed box* are expanded in the *inset* (**d**). In each panel, a wedge above the trace shows the period when the microelectrode was moved forward or backward. **e**–**g** In silico assays using the fi-NHK model. Traces show the simulated results on EP (*orange*; **e**), membrane potential on the basolateral surface of the syncytial layer, and its intracellular [K^+^] (*v*_SB_ and [K^+^]_SY_; **f**), and the potential and [K^+^] in the IS (ISP and [K^+^]_IS_; **g**) under normal conditions and during the blockade of syncytial Na^+^,K^+^-ATPases (*bars* above the traces). In the latter situation, the activity of ATPases was reduced to 46% of the initial value. In **e**–**g**, the initial values of all the potentials and concentrations were steady-state values that developed during 600 s after the onset of the simulation (for all the values, see Supplementary Table 2). The [K^+^]_IS_ behavior indicated by a *dashed box* is shown in the *inset* (**g**). In **g**, the simulated ISP dynamics (*red*) were compared to the change in the sum of the membrane potential across the syncytial basolateral surface (*v*_SB_) and a K^+^ equilibrium potential across the syncytial apical surface (*E*_K,SA_) (*green*). The *v*_SB_ values were derived from the simulation data described in **f**. *E*_K,SA_ was calculated by means of the Nernst equation *E*_K_ = RT/F([K^+^]_o_/[K^+^]_i_), where [K^+^]_o_ and [K^+^]_i_ are intracellular and extracellular [K^+^] adjacent to the membrane, with the [K^+^]_SY_ and [K^+^]_IS_ values simulated in the model **f**, **g**. **h**–**j** Comparison of the simulated and experimental data. The *panels* show values of endocochlear potential (EP; **h**), the membrane potential of the syncytial basolateral surface and syncytial [K^+^] (*v*_SB_ and [K^+^]_SY_, respectively; **i**), and the potential and [K^+^] of the IS (ISP and [K^+^]_IS_, respectively; **j**). *Open squares* indicate simulated steady-state values under the normal conditions and under conditions of blockage of syncytial-Na^+^,K^+^-ATPases (*left* and *right* in each *panel*, respectively). Under the latter conditions, *κ*_Ouabain,FC_ was set to 0.46. *Filled circles* are steady-state values obtained by experimental measurements during perilymphatic perfusion with a control solution and a solution containing 10 µM ouabain (*left* and *right* in each *panel*, respectively); a set of these two data types was obtained from each individual cochlea we examined, and therefore they are connected by lines in the respective *panel*. Of note, in three cochleae, all the compartments in the lateral wall were successfully captured by K^+^-selective microelectrodes, whereas in one cochlea, only the data on EP, ISP, and [K^+^]_IS_ were acquired. All the displayed values are the same as the individual simulated and experimental data used for the analyses in Table 1

While EP was continuously monitored with the glass microelectrode located in the endolymph of the scala media, the potential and [K^+^] in the lateral wall were recorded by double-barreled microelectrodes sensitive to the [K^+^] and potential, namely K^+^-selective microelectrodes. For measurements of potentials, perilymph always corresponded to 0 mV by our definition. Figure 3a–d shows the representative data obtained from a cochlea. Under physiological conditions, EP was +83.9 mV (Fig. 3a). This value changed only minimally during the perfusion with control artificial perilymph. To simultaneously analyze the syncytial layer, a K^+^-selective microelectrode continued to be advanced from perilymph to the IS (Fig. 3b) because it is difficult to hold the electrode inside this compartment, which comprises morphologically infolded and complicated fibrocytes.^28–30, 49^ At the outset, the K^+^-selective microelectrode in perilymph recorded [K^+^] of 5.1 mM. On the pathway toward the IS, spikelike increases of [K^+^] were detected along with synchronized mild increases in the potential. These data indicated that the electrode passed across multiple fibrocytes constituting the basolateral surface of the syncytial layer, as reported elsewhere.^28–30^ Then, the electrode encountered the IS showing low [K^+^], 7.3 mM, and a highly positive potential: +61.0 mV.^13, 15, 41^ Our previous studies revealed that among the aforementioned multiple compartments within the syncytial layer, the one immediately prior to the IS has the highest and stable values of [K^+^] and potential, and therefore it represents the properties of the layer.^28–30^ According to this criterion, in the experiment shown in Fig. 3b, syncytial [K^+^] (i.e., [K^+^]_SY_) and potential (i.e., *v*_SB_) were found to be 96.3 mM and +5.7 mV, respectively. During the subsequent recording, while holding the same K^+^-selective microelectrode in the IS, we applied a solution containing 10 µM ouabain to the perilymphatic space of the scala tympani (Fig. 3d). ISP was gradually hyperpolarized with time constants of *τ* = 1031.3 s and reached a plateau of +14.7 mV approximately 40 min after the onset of the perfusion. Careful observations clearly revealed that [K^+^] in the IS ([K^+^]_IS_) was reduced moderately during the perfusion (Fig. 3d, *inset*). EP decayed in a manner similar to that of ISP (*τ* = 1006.3 s; Fig. 3a). When the EP reduction stabilized, the K^+^-selective microelectrode was moved forward to endolymph; it recorded +6.2 mV (Fig. 3d). This potential resembled the measurement by the other electrode that had been placed in endolymph (+8.0 mV; Fig. 3a), confirming the accuracy of our experiments. After withdrawal to perilymph (Fig. 3d), a K^+^-selective microelectrode was again inserted from perilymph toward endolymph to evaluate syncytial properties during the perfusion with ouabain as shown in Fig. 3c (see also Fig. 3a). *v*_SB_ and [K^+^]_SY_, which represented the properties of the compartment immediately prior to the IS, were −4.3 mV and 11.7 mM, respectively. These results indicate that ouabain reduced both *v*_SB_ and [K^+^]_SY_, as compared with the data during application of the control solution (Fig. 3b). After farther insertion of the K^+^-selective electrode (Fig. 3c), we detected the IS with a potential of +10.9 mV. This value was almost identical to the EP value recorded by the other electrode in endolymph (+8.2 mV; Fig. 3a, c). It indicates that ouabain barely modified the voltage difference across the marginal-cell layer.

Using the same procedures as in Fig. 3a–d, we examined 28 cochleae; only in three cases did we clearly analyze all the compartments of the lateral wall, and additionally, in one trial, we could evaluate the IS properties but could not stably detect the syncytial layer. In all these measurements, the effects of ouabain on EP stabilized at approximately 40 min after the onset of its perfusion. Averaged values of EP and the potentials and K^+^ concentrations of the lateral-wall compartments at 40 min after the onset of perfusion with ouabain as well as those at 5 min before the onset are shown in Table 1. Thus, the perfusion with 10 µM ouabain reduced all of the following: EP, *v*_SB_, [K^+^]_SY_, ISP, and [K^+^]_IS_. We also tested 50 µM ouabain. Out of 24 cochleae we examined, in four cochleae, we succeeded in measuring all the five values mentioned above. The results were similar to those obtained with 10 µM ouabain (Supplementary Table 3).

In live guinea pigs, the perfusion with ouabain seems to dysfunction Na^+^,K^+^-ATPases on the syncytial basolateral surface directly exposed to perilymph.^28, 50^ Under physiological conditions, ISP resembles EP; therefore, the voltage difference across the marginal-cell layer is small.^13, 15, 17^ When Na^+^,K^+^-ATPases on the basolateral surface of marginal-cell layer are inhibited by any perturbation, [K^+^] in this layer ([K^+^]_MC_) is reduced, and thus *E*_K_ on its apical surface is enhanced.^15^ As a consequence, the transepithelial voltage across the marginal-cell layer increases. These changes may account for the induction of a significant difference between ISP and EP by perilymphatic perfusion of ouabain at 1 mM.^28^ In these contexts, during the application of ouabain at 10 µM, the difference between ISP and EP was small (several millivolts) even when the effects of the drug were maximized and stabilized (see Fig. 3c, d). This observation indicates that the perturbation seemed to minimally affect the marginal-cell Na^+^,K^+^-ATPases. Accordingly, we intended to simulate the experimental condition of 10 µM drug perfusion using the fi-NHK model by blocking only the syncytial Na^+^,K^+^-ATPases. Because the degree of this blockage is unmeasurable in vivo, in the model, we used an estimated value. When the activity of syncytial Na^+^,K^+^-ATPases was reduced to 46% of their normal value (i.e., blocking rate *κ*_Ouabain,FC_ = 0.46), all the simulated steady-state values of EP, *v*_SB_, [K^+^]_SY_, ISP, and [K^+^]_IS_ fell approximately within the range of the experimental data obtained during perfusion with 10 µM ouabain (Supplementary Fig. 2a) and resembled their averaged values (Table 1). The dynamics of these parameters are displayed in Fig. 3e–g. Under normal conditions, EP, *v*_SB_, and [K^+^]_SY_ were +72.7 mV, +9.6 mV, and 98.3 mM, respectively (Fig. 3e, f); the IS showed a potential of +81.2 mV (ISP) with [K^+^] of 6.1 mM ([K^+^]_IS_; Fig. 3g). As shown in Fig. 3f, we reconstituted the conditions of ouabain perfusion; *v*_SB_ gradually hyperpolarized with a time constant of *τ* = 240.0 s and stabilized in 25 min. [K^+^]_SY_ declined more slowly (*τ = *288.2 s; Fig. 3f). EP, ISP, and [K^+^]_IS_ decayed in a similar manner (*τ = *454.4, 453.6, and 454.1 s, respectively) and reached a plateau in ~30 min (Fig. 3e, g). In Fig. 3h–j, the measured steady-state values of EP and the potential and [K^+^] in the syncytial layer and IS in individual experiments were plotted and compared to the simulated steady-state values; these two lists of data were comparable with each other. Taken together, our simulations did not completely replicate time course data on the responses of our in vivo assays but reasonably mirrored overall observations (Fig. 3a–d). It is noteworthy that the measured change of [K^+^]_IS_ (Fig. 3d) was reasonably reproduced by the model (Fig. 3g; see also Fig. 3j and Table 1) although the IS is not adjacent to the syncytial basolateral surface harboring Na^+^,K^+^-ATPases, which were blocked in the simulation (see Figs. 1b and 2c). These results confirm the assumption of the fi-NHK model.

Experimental approaches have previously indicated that ISP, a major component of EP, depends on *E*_K_ produced on the apical surface of the syncytial layer^9, 13–15, 20, 21^:

13 $${\rm{ISP}} = {v_{{\rm{SB}}}} - {v_{{\rm{SA}}}} \approx {v_{{\rm{SB}}}} - \frac{{RT}}{F}{\rm{ln}}\left( {\frac{{{{\left[ {{{\rm{K}}^ + }} \right]}_{{\rm{IS}}}}}}{{{{\left[ {{{\rm{K}}^ + }} \right]}_{SY}}}}} \right).$$

For further evaluation of our updated model, we tested whether this characteristic could be reproduced in silico. ISP was calculated using Eq. (13) and the *v*_SB_, [K^+^]_SY_, and [K^+^]_IS_ values obtained in the fi-NHK model during the inhibition of syncytial Na^+^,K^+^-ATPases (Fig. 3f, g). These ISP values were overlaid with the ISP values simulated directly by the model (Fig. 3g). The two traces matched well, reinforcing the validity of the model. Therefore, the circulation current across the syncytial basolateral surface is likely to depend primarily on Na^+^,K^+^-ATPases, Na^+^ conductance, and leak conductance.

### Relevance of the syncytial ion transport mechanisms to the circulation current

**Fig. 4:**
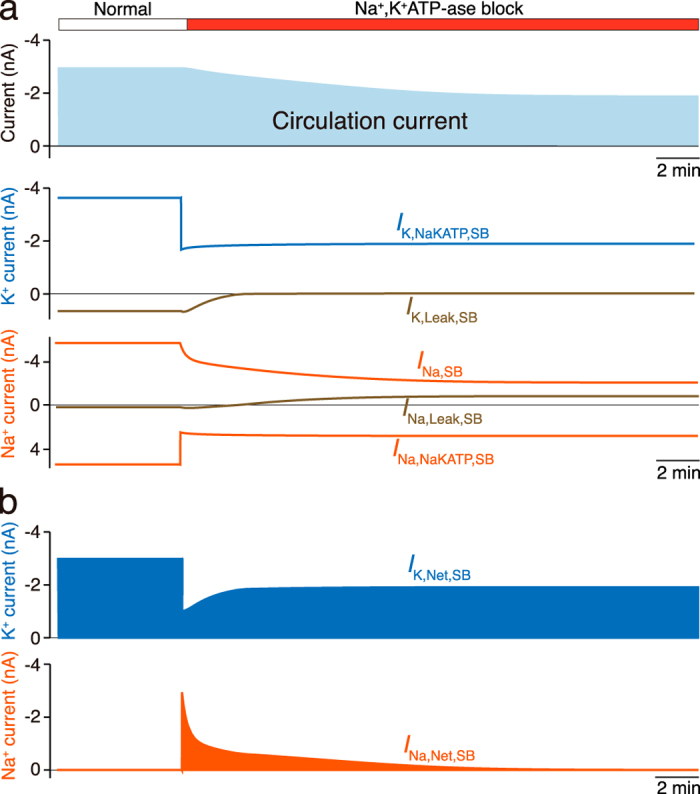
Simulated dynamics of the current across the syncytial basolateral surface. **a** The circulation current and the K^+^ and Na^+^ currents through Na^+^,K^+^-ATPases, through leak conductance, and through Na^+^ conductance on the syncytial basolateral surface (SB) under normal conditions and during blockade of Na^+^,K^+^-ATPases (46% of the initial activity) were simulated and are displayed in the *upper*, *middle*, and *bottom panels*, respectively. **b** Illustrated in *upper* and *lower panels* are the net K^+^ and Na^+^ currents across the syncytial basolateral surface, respectively. Each current represents a sum of the K^+^ or Na^+^ flow conveyed through all the conductances and transporters on the membrane (Supplementary Fig. 3)

We finally used the fi-NHK model to study how the three ion transport machineries on the syncytial basolateral surface participate in the establishment of the circulation current. Figure 4a illustrates the dynamics of the circulation current and Na^+^ and K^+^ flows through each ion transport machinery on the syncytial basolateral surface (see also Supplementary Fig. 3). Under normal conditions, the circulation current, which consists solely of K^+^ in any compartments, equals the sum of all the ionic currents in each membrane domain. On the syncytial basolateral surface, the circulation current is occupied mainly by the K^+^ flow of Na^+^,K^+^-ATPases. This phenomenon stems from the arrangement where Na^+^ flow is canceled out by Na^+^ recycling among the three ion transport machineries, i.e., the ATPases, Na^+^ conductance, and leak conductance (Fig. 4a; see also Eqs. (10–12) and Fig. 2d). When the activity of syncytial Na^+^,K^+^-ATPases was reduced to 46% of its normal value, outward Na^+^ flow and inward K^+^ flow through the ATPases rapidly and concomitantly decreased (Fig. 4a). In response to this perturbation, the circulation current declined more slowly, with a time constant of *τ = *467.9 s, and stabilized at −1.9 nA in ~30 min. Ionic flows through the leak conductance showed complicated behaviors. During the perturbation, the outward K^+^ flow decreased almost to zero with a time constant of *τ* = 89.2 s. On the other hand, the Na^+^ flow, which had initially been outward, switched to the inward direction at ~3 min after the onset of the blockage. Furthermore, the current through Na^+^ conductance was gradually suppressed with a time constant of *τ* = 169.6 s. These kinetics were slower than the change in Na^+^ flow via ATPases and more rapid than the change in the circulation current (*τ* = 467.9 s). We next calculated the net K^+^ and Na^+^ currents carried by all the ion transport machineries (Fig. 4b; see also Supplementary Fig. 3). Under normal conditions, the circulation current consisted only of K^+^. At the onset of the perturbation, the inward K^+^ current abruptly decreased. Simultaneously, a remarkable Na^+^ current emerged. Thereafter, the K^+^ current partially recovered, whereas the Na^+^ current gradually decreased toward the initial level. Finally, in the steady state, the net Na^+^ current disappeared; therefore, the circulation current was again governed completely by the K^+^ flow. These observations indicate that a fraction of the circulation current during its reduction phase was transiently switched from K^+^ to Na^+^ across the syncytial basolateral surface. Furthermore, throughout the perturbation, the amplitude of the outward Na^+^ current via Na^+^,K^+^-ATPases remained approximately constant (2.5–2.8 nA, Fig. 4a). Accordingly, the changed behaviors of the currents through the Na^+^ and leak conductances are involved in the transiently evoked Na^+^ inflow. This finding also confirms the contribution of these ion transport machineries to the circulation current.

## Discussion

Using the fi-NHK model, here we demonstrated that establishment and a change of the circulation current across the syncytial basolateral surface depend primarily on the Na^+^ and leak conductances and Na^+^,K^+^-ATPases. This achievement results in determination of the whole system driving the circulation current. Again, abundance of Na^+^ conductance is relatively unusual, because in general, cell membranes are dominated by K^+^ or Cl^−^ conductance in the resting state^31–33^ (see Introduction). Nonetheless, our simulations implied that this profile is necessary for the electrochemical homeostasis in the cochlea. On the syncytial basolateral surface, the behavior of Na^+^ plays key roles in ionic flow. Under normal conditions, the net Na^+^ current is zero because this ion locally recycles among the three ion transport machineries on the syncytial basolateral surface (Figs. 2d and 4b). In contrast, when the Na^+^,K^+^-ATPases are blocked, the currents through the two conductances are modulated in different ways, contributing to the emergence of Na^+^ inflow as net flow (Fig. 4). This Na^+^ flow and the reduced net K^+^ inflow contribute to the circulation current as well as to changes in syncytial ion concentrations underlying *v*_SB_ hyperpolarization (Figs. 3f and 4; Supplementary Fig. 2b, c). In vivo, neither currents via channels and transporters nor ionic flows across the membranes are measurable by electrophysiological methods. Computational modeling using the fi-NHK model is an effective way to address the topics related to the circulation current.

Perilymphatic perfusion with 50 μM ouabain significantly reduced [K^+^]_IS_ in this study, where we used an improved experimental technique (Supplementary Table 3), but previously, this perturbation had little or no effect on [K^+^]_IS_.^28, 29^ The reason for this discrepancy may be the following. In our earlier work, larger fenestrae for insertion of the microelectrodes were made on the cochlear bony wall.^28, 29^ Membranes of some lateral-wall cells may have been somehow injured and could have become fragile during the surgical procedure. Therefore, when the electrodes were advanced, [K^+^] could have been leaked to the narrow IS from inside the cells. This event could compensate the [K^+^]_IS_ decrease induced by inhibition of the syncytial Na^+^,K^+^-ATPase. Further studies will be necessary to test the above hypothesis.

Steady-state values of EP and various electrochemical profiles of the lateral wall during application of 10 μM ouabain resembled those during application of 50 μM ouabain (Supplementary Table 3). Therefore, the effects on the cochlea were likely to be saturated at the ouabain concentration of 10 μM. In vitro experiments indicated that 10 μM ouabain suppressed more than 90% of the activity of Na^+^,K^+^-ATPases composed of α_1_ and β_1_ subunits (IC_50_ = 2 μM),^51^ which are assembled in cochlear fibrocytes as well.^25^ This evidence allows us to hypothesize that in vivo, a population of syncytial ATPase molecules, which was directly exposed to the artificial perilymph containing ouabain at 10 or 50 μM, was blocked to a similar degree and functioned only minimally at either concentration of ouabain. Nevertheless, the experimental measurements were reasonably reproduced in the fi-NHK model when the activity of the ATPases was reduced to 46% of the normal value (Fig. 3 and Table 1; Supplementary Fig. 2a). There are two scenarios to explain the validity of the blocking rate in the simulation. The first possibility is related to the morphology and distribution of the fibrocytes that constitute the syncytial basolateral surface. The fibrocytes harbor highly invaginated membranes and are tightly wrapped with connective tissue including collagen.^49^ Moreover, the cells are scattered in the spiral ligament.^49, 52^ Because of these characteristics, the perfused artificial perilymph containing ouabain could reach a limited number of the fibrocytes or a limited region of their membrane. In other words, the number of Na^+^,K^+^-ATPase molecules fully blocked with 10 μM ouabain may be similar to that with 50 μM ouabain and the rest of the transporters in both cases may be almost unaffected. Of note, in the present study, this condition was reproduced by changing total Na^+^,K^+^-ATPase activity of each fibrocyte by the same factor: 0.46 (i.e., the determined blocking rate; see Model development and Supplementary Information), because the aforementioned configuration of fibrocytes and Na^+^,K^+^-ATPases, which has not yet been quantitatively determined by a histological approach, is not incorporated into the fi-NHK model. Alternatively, unidentified ion transport machineries in the fibrocytes could partially cancel the electrochemical change induced by the complete blockade of Na^+^,K^+^-ATPases.

In Fig. 3, the time course of the reduction in EP and ISP in the experiment was slower than that in the simulation. This difference may be caused by only a rough estimate of the volumes of the intracellular and extracellular compartments in the model or the aforementioned histological profile of the fibrocytes. Furthermore, to replace all native perilymph with the artificial one (8.9 µl in a cochlea) using our perfusion system (speed: 10 µl/min), approximately 1 min would be required.^53^ The perfusion speed would also be reduced by the flow of native perilymph.^54^ Tuning the kinetics for inhibition of Na^+^,K^+^-ATPase in the model will be mandatory to simulate the experimental observations more precisely.

## Methods

### In vivo electrophysiological recordings

Ethical statement about animal use and approval of the experimental protocol are the same as the ones described in the section “Morphometry of the tissues in the lateral cochlear wall.” Each experiment was conducted during the light phase. Male Hartley guinea pigs (200–400 g, 3–5 weeks of age; SLC Inc.), whose hearing level was determined with normal Preyer’s reflex, were intraperitoneally administrated with pentobarbital sodium (64.8 mg/kg). The corneal reflex, toe pinch, and respiratory rate were examined to evaluate the depth of anesthesia. When anesthesia was insufficient, the drug (5 mg/kg) was added into the guinea pigs. Next, the animals were treated intramuscularly with vecuronium bromide (4 mg/kg) and were artificially ventilated with room air. Throughout the assays, the animals were kept at 37°C using a heater (blanket type; BWT-100A, Bio Research Center, Nagoya, Japan). The heart rate was monitored to assess the depth of anesthesia. During each experiment, pentobarbital sodium (10 mg/kg) was added to the animal every 1–1.5 h. After termination of the recordings, the animals were euthanized with an excess amount of the anesthesia (400 mg/kg).

The cochleae of living guinea pigs were electrophysiologically examined using a procedure similar to that in our earlier reports.^15, 28, 29^ The details are described in Supplementary Information. A measurement was assumed to be successful when it satisfied both of the following conditions; first, the EP value recorded in physiological settings exceeded +60 mV, and second, voltage drift observed when the glass microelectrode was pulled back to perilymph was less than ± 5 mV. Randomization and blinding of the experimental groups were not required.

### Statistics

Mean ± standard deviation served as a descriptive statistic. No statistical analyses were performed to predetermine sample sizes, but our sample sizes are similar to those generally employed in the field. ^13, 15, 28–30, 41^ Because of the low success rate of the experiments, ^13, 15, 28–30, 41^ measured values in a group were empirically estimated to follow a normal distribution. The distributions of the measured values among groups were compared using the paired *t* test and unpaired *t* test as shown in Table 1 and Supplementary Table 3, respectively. Data with a *p* value < 0.05 were considered significant. All the statistical analyses were carried out using GraphPad Prism 7 (GraphPad Software, Inc., CA, USA).

### Data availability

Supplementary Information includes a detailed description of the model, definitions and abbreviations, formulations of the model, experimental procedures, three figures, and three tables. All data supporting the findings of this study are available within the paper and Supplementary Information. All custom MATLAB code (fibrocyte integrated NHK model version X) used in the simulation and Supplementary Information are freely available at https://doi.org/10.6084/m9.figshare.5188681.v1 and on the *NPJ Systems Biology and Applications* website, respectively.

## Electronic supplementary material

Supplementary Information

Supplementary Figure 1

Supplementary Figure 2

Supplementary Figure 3

## Electronic supplementary material

**Supplementary Information** accompanies the paper on the *npj Systems Biology and Applications* website (doi:10.1038/s41540-017-0025-0).
