# Author Correction: Computer modeling defines the system driving a constant current crucial for homeostasis in the mammalian cochlea by integrating unique ion transports

**DOI:** 10.1038/s41540-021-00197-3

**Published:** 2021-10-18

**Authors:** Fumiaki Nin, Takamasa Yoshida, Shingo Murakami, Genki Ogata, Satoru Uetsuka, Samuel Choi, Katsumi Doi, Seishiro Sawamura, Hidenori Inohara, Shizuo Komune, Yoshihisa Kurachi, Hiroshi Hibino

**Affiliations:** 1 Department of Molecular Physiology, Niigata University School of Medicine, Niigata, Japan; 2 Center for Transdisciplinary Research, Niigata University, Niigata, Japan; 3 AMED-CREST, AMED, Niigata, Japan; 4 Department of Otorhinolaryngology, Graduate School of Medical Sciences, Kyushu University, Fukuoka, Japan; 5 Department of Physiology, School of Medicine, Toho University, Tokyo, Japan; 6 Department of Otorhinolaryngology–Head and Neck Surgery, Graduate School of Medicine, Osaka University, Suita, Japan; 7 Department of Electrical and Electronics Engineering, Niigata University, Niigata, Japan; 8 Department of Otolaryngology, Faculty of Medicine, Kindai University, Osakasayama, Japan; 9 Division of Otolaryngology–Head and Neck Surgery, Yuaikai Oda Hospital, Kashima, Japan; 10 Division of Molecular and Cellular Pharmacology, Department of Pharmacology, Graduate School of Medicine, Osaka University, Suita, Japan; 11 The Global Center for Medical Engineering and Informatics, Osaka University, Suita, Japan

**Keywords:** Physiology, Multicellular systems, Computer modelling

Correction to: *npj Systems Biology and Applications* 10.1038/s41540-017-0025-0, published online 25 August 2017

In Fig. 2a of this article an inaccurate equation was shown for the circulation current; the equation should have appeared as shown below. This has now been amended in the HTML and PDF versions of the article.

**Figure:**
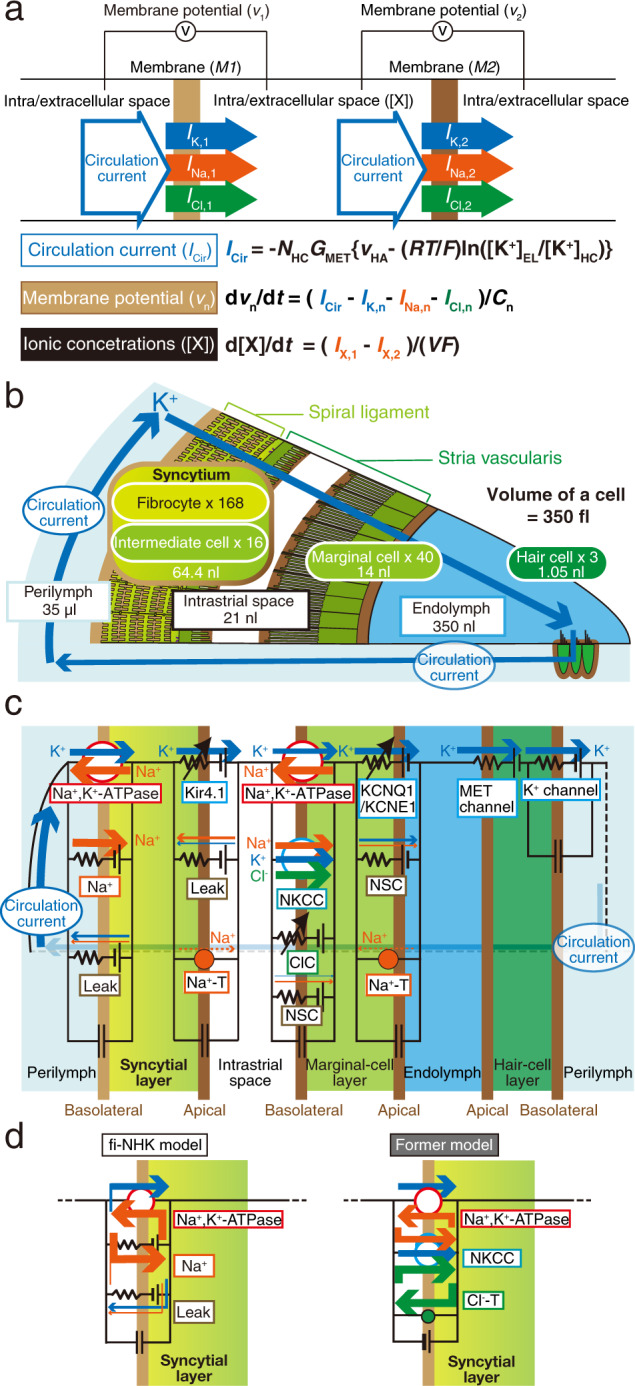
Fig. 2

In the Supplementary Information for this article, the equations e, i and j in section IV. Ionic flows and currents were incorrect. The descriptions of *n* and *T* were incorrect, and *n*_∞_ was not described. A new Supplementary Information file has been uplodaded with the correct information.

## Supplementary information

Supplementary Information

