# Supplementary figures and images for: Computer modeling defines the system driving a constant current crucial for homeostasis in the mammalian cochlea by integrating unique ion transports
Source: NPJ Syst Biol Appl. 2017 Aug 25;3:24. doi: 10.1038/s41540-017-0025-0 (PMC5572463; doi:10.1038/s41540-017-0025-0)

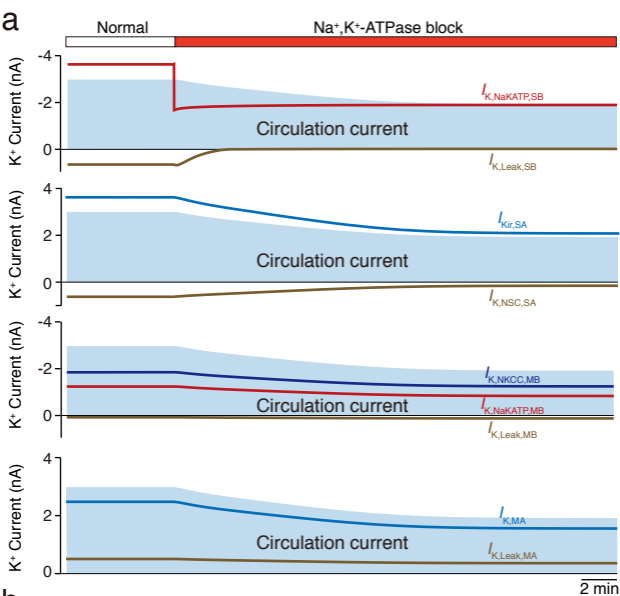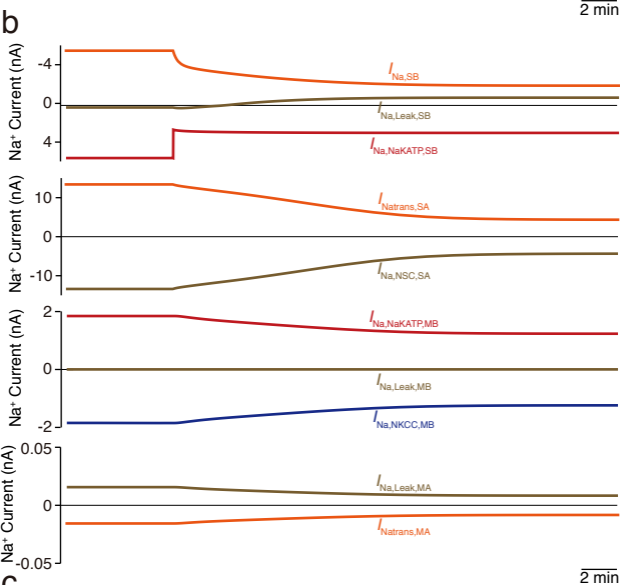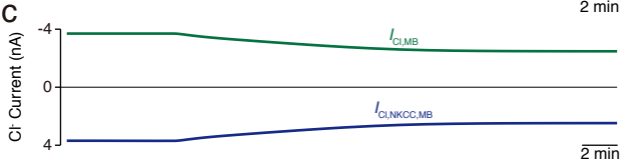

Supplement: Supplementary file 3 — Supplementary Figure 2 [file 41540_2017_25_MOESM3_ESM.pdf]

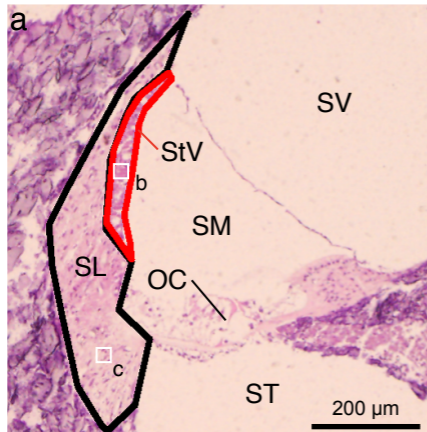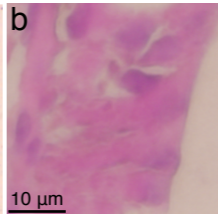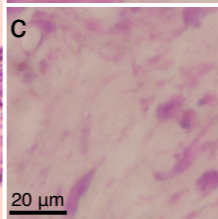

Supplement: Supplementary file 4 — Supplementary Figure 3 [file 41540_2017_25_MOESM4_ESM.pdf]
