# Supplementary material for: Computer modeling defines the system driving a constant current crucial for homeostasis in the mammalian cochlea by integrating unique ion transports
Source: NPJ Syst Biol Appl. 2017 Aug 25;3:24. doi: 10.1038/s41540-017-0025-0 (PMC5572463; doi:10.1038/s41540-017-0025-0)
Supplement: Supplementary file 2 — Supplementary Figure 1 [file 41540_2017_25_MOESM2_ESM.pdf]

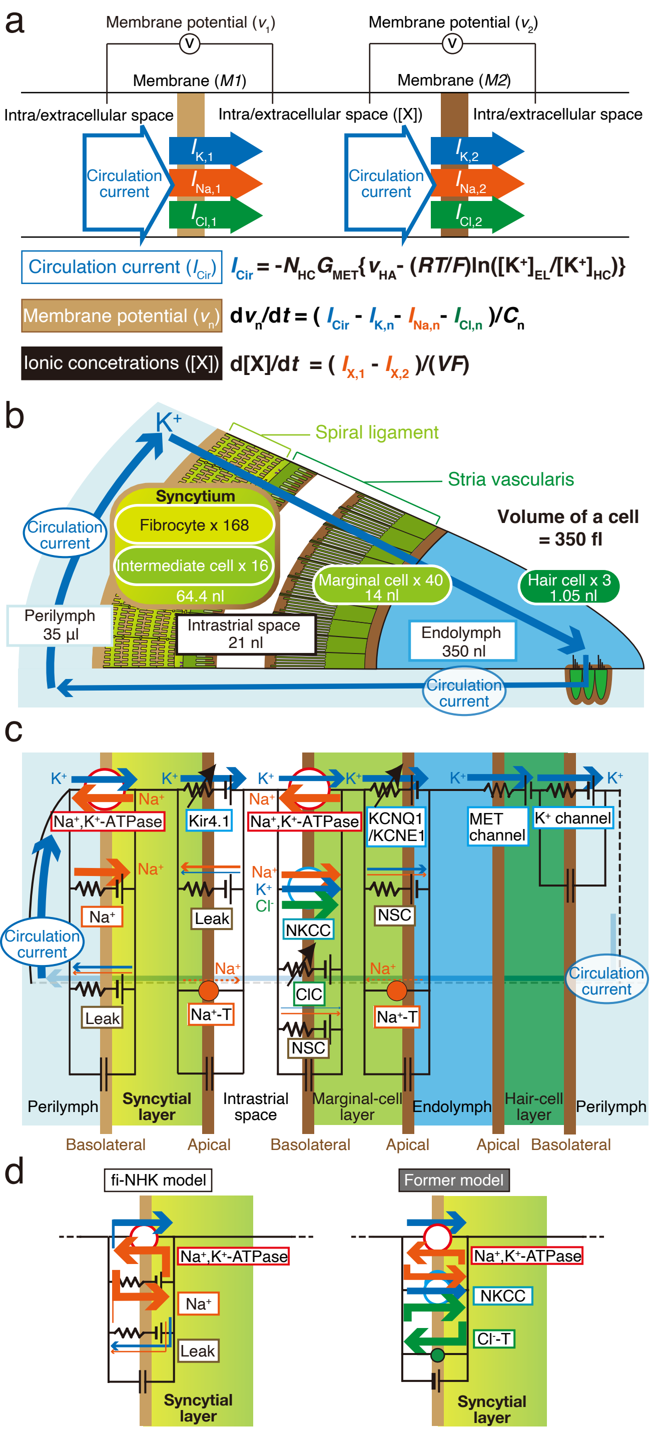

a
Membrane potential (v_1)
Membrane potential (v_2)
Membrane ($M1$)
Membrane ($M2$)
Intra/extracellular space
Intra/extracellular space ([X])
Intra/extracellular space
Circulation current
$I_{K,1}$
$I_{Na,1}$
$I_{Cl,1}$
Circulation current
$I_{K,2}$
$I_{Na,2}$
$I_{Cl,2}$
Circulation current (I_{Cir})
$I_{Cir} = -N_{HC}G_{MET}\{v_{HA} - (RT/F)\ln([K^+]_{EL}/[K^+]_{HC})\}$
Membrane potential (v_n)
$dv_n/dt = (I_{Cir} - I_{K,n} - I_{Na,n} - I_{Cl,n})/C_n$
Ionic concetrations ([X])
$d[X]/dt = (I_{X,1} - I_{X,2})/(VF)$
b
K^+
Spiral ligament
Stria vascularis
Syncytium
Fibrocyte x 168
Intermediate cell x 16
64.4 nl
Volume of a cell = 350 fl
Circulation current
Marginal cell x 40
14 nl
Hair cell x 3
1.05 nl
Perilymph
35 µl
Intrastrial space
21 nl
Endolymph
350 nl
Circulation current
c
K^+
Na^+
Na^+,K^+-ATPase
Kir4.1
KCNQ1 /KCNE1
MET channel
K^+ channel
Na^+
Leak
Na^+
K^+
Cl^-
NKCC
NSC
Circulation current
ClC
Na^+-T
NSC
Leak
Perilymph
Syncytial layer
Intrastrial space
Marginal-cell layer
Endolymph
Hair-cell layer
Perilymph
Basolateral
Apical
Basolateral
Apical
Apical
Basolateral
d
fi-NHK model
Former model
Na^+,K^+-ATPase
Na^+
Leak
NKCC
Cl^--T
Syncytial layer
Syncytial layer
