# Supplementary material for: Author Correction: Computer modeling defines the system driving a constant current crucial for homeostasis in the mammalian cochlea by integrating unique ion transports
Source: NPJ Syst Biol Appl. 2021 Oct 18;7:39. doi: 10.1038/s41540-021-00197-3 (PMC8523537; doi:10.1038/s41540-021-00197-3)
Supplement: Supplementary file 1 — Supplementary Information [file 41540_2021_197_MOESM1_ESM.pdf]

Supplementary Information

Supplementary Methods

1. Detailed description of the fi-NHK model

The computational model used in this study is an updated version of the NHK (Nin-Hibino-Kurachi) model that we previously developed to reproduce the circulation current and endocochlear potential (EP). [1] The major revision as well as the model's principles, which are mostly identical to those of the former model, are described below. The model was designed to reproduce the electrochemical phenomena of the cochlea in 10-µm slices, a distance that is comparable to the width of one row of hair cells and lateral wall cells. The amplitudes and characteristics of EP and of the circulation current were inferred from the function of ion conductance and transporters as well as the morphological features of the cochlea, which includes three layers, i.e., syncytial, marginal-cell, and hair-cell layers, and three extracellular spaces, i.e., the intrastrial space (IS), perilymph, and endolymph (**Figure 2b** and **2c**).

IS potential (ISP), marginal-cell potential (MCP), and EP represent different combinations of membrane potentials in the lateral wall[1,2] (**Equations S1–S3**):

$ISP = v_{SB} - v_{SA}$, **Equation S1** (same as **Equation 2**)

$MCP = v_{SB} - v_{SA} + v_{MB}$, **Equation S2**

$EP = v_{SB} - v_{SA} + v_{MB} - v_{MA}$, **Equation S3** (same as **Equation 1**)

where v_{SB}, v_{SA}, v_{MB}, and v_{MA} are the membrane potentials across the basolateral and apical surfaces of the syncytial layer and through basolateral and apical surfaces of the marginal-cell layer, respectively. Membrane potentials are relative to the neighboring extracellular fluid, which corresponds to 0 mV by our definition. ISP, MCP, and EP, which are potentials inside the IS, marginal cells, and endolymph, respectively, are shown in reference to perilymph (**Figure 1b**).

The circulation current was predicted to flow in a closed-loop circuit that crosses the two layers of the lateral wall and the hair-cell layer. In each membrane domain, ion conductance and transporters shown in **Figure 2c** help to drive the circulation current. They were extracted from immunohistochemical and electrophysiological data.[3,4] The key players involved in the regulation of EP and of the circulation current include Na^+,K^+-ATPases on the fibrocyte membrane;[5-7] Kir4.1 K^+ conductance on the intermediate-cell membrane;[8,9] Na^+,K^+,$2Cl^-$-cotransporters NKCC, Na^+,K^+-ATPases, and ClC/K Cl^- conductance on the basolateral membrane of marginal cells;[10-12] and KCNQ1/KCNE1 K^+ conductance on the apical membrane of marginal cells.[13,14] Mechanoelectrical

transduction (MET) channels were considered a sole pathway for the circulation current (I_{Cir}) across the apical surface of the hair-cell layer (**Equation S4**):

$$I_{Cir} = -N_{HC} \cdot I_{MET} \qquad \textbf{Equation S4}$$

where N_{HC} is the number of hair cells, and I_{MET} is the total MET current in a hair cell.

The amplitude of the MET current likely depends on the potential difference across the apical surface of the hair cell.[15] Additionally, K^+ appears to predominantly permeate the MET channels *in vivo*.[16] These arrangements are given by the following two equations:

$$v_{HA} = -EP + v_{HB} \qquad \textbf{Equation S5} \text{ (same as } \textbf{Equation 3}\text{)}$$

$$I_{MET} = G_{MET}\left(v_{HA} - \frac{RT}{F} \cdot ln\left(\frac{[K^+]_{EL}}{[K^+]_{HC}}\right)\right) \qquad \textbf{Equation S6} \text{ (same as } \textbf{Equation 4}\text{)}$$

where $[K^+]_{EL}$ and $[K^+]_{HC}$ are $[K^+]$ in endolymph and in the hair cell, respectively; G_{MET} is the conductance of MET channels; v_{HA} and v_{HB} denote the membrane potentials of a hair-cell's apical and basolateral surfaces, respectively; R is the gas constant, T represents temperature, and F is the Faraday constant. Notably, the MET current exists even without acoustic stimuli (~1 nA/cell).[17,18]

In the model, the difference between I_{Cir}, which flows into a membrane, and I_M, a net current that results from the sum of ionic flows through all the ion conductances and transporters on the same membrane, directly contributes to the change in membrane potential, v:

$$\frac{dv}{dt} = \frac{1}{C}\frac{dQ}{dt} = \frac{I_{Cir} - I_M}{C} = \frac{I_{Cir} - I_K - I_{Na} - I_{Cl}}{C} \qquad \textbf{Equation S7} \text{ (same as } \textbf{Equation 5}\text{)}$$

where C is capacitance, Q is the charge accumulated on the membrane, and I_K, I_{Na}, and I_{Cl} represent K^+, Na^+, and Cl^- current fractions constituting I_M, respectively.

Furthermore, the change in ionic concentration, $[X^+]$, in six extracellular and intracellular spaces depends on the difference between the inward and outward currents of X^+ ($I_{X,In}$ and $I_{X,Out}$, respectively):

$$\frac{d[X^+]}{dt} = \frac{I_{X,In} - I_{X,Out}}{V \cdot F} \qquad \textbf{Equation S8} \text{ (same as } \textbf{Equation 6}\text{)}$$

where V is the volume of the intra- or extracellular space and was set to be constant (**Figure 2b** and **Supplementary Table 1**). Note that $I_{X,In}$ and $I_{X,Out}$ are constituents of I_M. When EP is in the steady state, all membrane potentials and ionic concentrations are stable so that I_{Cir} corresponds to I_M, and $I_{X,In}$ equals $I_{X,Out}$ (**Equations S7** and **S8**). These relations are also applicable to any anions.

The number of fibrocytes was determined by histological analysis as described in the **Model development** of the main text (see the section "***Morphometry of the tissues in the lateral cochlear wall***"). In the model, we proposed that all cell types in the lateral wall and hair cells have equal

volume (see **Figure 2b** and **Supplementary Table 1**).[19] Basal cells have very thin cell bodies.[20] Additionally, because these cells were tightly packed with intermediate cells and fibrocytes (**Figure 1b**),[21] the membrane area exposed to perilymph and the IS fluid is small. On the basis of these characteristics, we omitted the contribution of basal cells to electrochemical properties of the lateral wall in the model. Of note, because the basolateral surface of the syncytial layer is composed of multiple fibrocytes, the total current through this membrane domain (I_{SB}) and its capacitance (C_{SB}) are described as

$$I_{\mathrm{SB}} = N_{\mathrm{FC}} \cdot I_{\mathrm{FC}} \quad \textbf{Equation S9}$$

$$C_{\mathrm{SB}} = N_{\mathrm{FC}} \cdot C_{\mathrm{FC}} \quad \textbf{Equation S10}$$

where I_{FC} and C_{FC} are the current through the membrane of a fibrocyte and its capacitance, respectively, and N_{FC} denotes the number of the fibrocytes constituting the basolateral surface of the syncytial layer. C_{FC} was assumed to be identical to the capacitance of an intermediate cell because both cell types harbor a highly invaginated membrane and are morphologically similar (**Supplementary Table 1**).[2,8,21-23]

On the basis of **Equations S7–S10**, v_{SB} and ionic concentrations inside the syncytial layer ($[\mathrm{K}^+]_{\mathrm{SY}}$, $[\mathrm{Na}^+]_{\mathrm{SY}}$, and $[\mathrm{Cl}^-]_{\mathrm{SY}}$), both of which were set to be constant under any conditions in our earlier model,[1] were defined as follows in the present model (see **Equations 5** and **6**):

$$\frac{dv_{\mathrm{SB}}}{dt} = \frac{-I_{\mathrm{SB}} - I_{\mathrm{Cir}}}{C_{\mathrm{SB}}} = \frac{-N_{\mathrm{FC}} \cdot I_{\mathrm{FC}} - I_{\mathrm{Cir}}}{N_{\mathrm{FC}} \cdot C_{\mathrm{FC}}} \quad \textbf{Equation S11}$$

$$\frac{d[\mathrm{K}^+]_{\mathrm{SY}}}{dt} = -\frac{N_{\mathrm{FC}} \cdot I_{\mathrm{K,FC}} + N_{\mathrm{IC}} \cdot I_{\mathrm{K,IC}}}{(N_{\mathrm{FC}} + N_{\mathrm{IC}}) \cdot V_{\mathrm{Cell}} \cdot F} \quad \textbf{Equation S12}$$

$$\frac{d[\mathrm{Na}^+]_{\mathrm{SY}}}{dt} = -\frac{N_{\mathrm{FC}} \cdot I_{\mathrm{Na,FC}} + N_{\mathrm{IC}} \cdot I_{\mathrm{Na,IC}}}{(N_{\mathrm{FC}} + N_{\mathrm{IC}}) \cdot V_{\mathrm{Cell}} \cdot F} \quad \textbf{Equation S13}$$

$$\frac{d[\mathrm{Cl}^-]_{\mathrm{SY}}}{dt} = 0 \quad \textbf{Equation S14}$$

where N_{IC} is the number of intermediate cells constituting the syncytial apical surface; $I_{\mathrm{K,FC}}$, $I_{\mathrm{Na,FC}}$, $I_{\mathrm{K,FC}}$, and $I_{\mathrm{Na,IC}}$ are the K^+ and Na^+ currents through the membranes of a fibrocyte and an intermediate cell, respectively; and V_{Cell} is the volume of a cell (**Figure 2c** and **Supplementary Table 1**). The change in the potential and $[\mathrm{K}^+]$, $[\mathrm{Na}^+]$, and $[\mathrm{Cl}^-]$ in the other compartments were simulated as reported in our earlier study[1] (see also "**Formulation of the model**" described below). We assumed that the volume of the syncytial layer, which is the product of the number of cells constituting the layer (sum of N_{FC} and N_{IC}) and the volume of a cell (V_{Cell}), were constant (**Supplementary Table 1**). Therefore, $[\mathrm{K}^+]_{\mathrm{SY}}$, $[\mathrm{Na}^+]_{\mathrm{SY}}$, and $[\mathrm{Cl}^-]_{\mathrm{SY}}$ are controlled solely by ionic currents through the membranes.

As assumed in the previous model, in the fi-NHK model, the Na^+ current through leak conductance on the apical surface of the syncytial layer and nonselective cation (NSC) conductance on the apical surface of the marginal-cell layer were set to be cancelled out by Na^+ outflow through the concomitant Na^+ transporters (**Figure 2d**).[1] As a consequence, these two membrane domains, in addition to the apical and basolateral surfaces of the hair-cell layer, allow only for K^+ flow as net flow under any conditions, simplifying our model (**Figure 2c** and "**Formulation of the model**" described below). Moreover, as described in the ***main text***, we determined the values of Na^+ and leak conductance and activity of Na^+,K^+-ATPases on the basolateral surface of the syncytial layer by solving **Equations 10–12**, which represent local Na^+ recycling among the three ion transport machineries under physiological conditions. Initial values that were applied to start the simulations in the model were based on the experimental data as denoted in **Supplemental Table 2**; a hair-cell's ion concentrations were set to be similar to the measured values of an intermediate or marginal cell.[24,25] Finally, to simulate effects of perilymphatic perfusion of ouabain at 10 µM, we determined a blocking rate for Na^+,K^+-ATPases on the basolateral surface of the syncytial layer as shown in "**Model development**" in the **main text** (see the section "Parameter settings") and modulated the activity of the ATPases as described in "**Formulation of the model**" below (see subsection "e. Flow via Na^+,K^+-ATPase in fibrocyte: $I_{NaKATP,FC}$"). Other equations defining the fi-NHK model were taken from the previous model[1] and are listed in the section "**Formulation of the model**."

2. *In vivo* experimental arrangements

2-I. Electrophysiological recordings

The experimental protocol was approved by the Animal Research Committees of Niigata University School of Medicine. The experiments were carried out under the supervision of the Committees and in accordance with the Guidelines for Animal Experiments of Niigata University and the Japanese Animal Protection and Management Law. Male Hartley guinea pigs (200–400 g; 3-5 weeks, SLC Inc.), whose hearing level was confirmed by normal Preyer's reflex, were anesthetized with intraperitoneal injection of pentobarbital sodium (64.8 mg/kg; Somnopentyl; Kyoritsu Seiyaku, Tokyo, Japan). A toe pinch, the corneal reflex, and respiratory rate served as indicators to evaluate the depth of anesthesia. When anesthesia was not sufficient, pentobarbital sodium (5 mg/kg) was additionally injected into the animals. Next, the animals were injected intramuscularly with the muscle relaxant vecuronium bromide (4 mg/kg) and were artificially ventilated with room air. Throughout the experiments, the body temperature of the animals was maintained at 37°C using a heating blanket (BWT-100A, Bio Research Center, Nagoya, Japan). The depth of anesthesia was

assessed by fluctuations in the heart rate. Anesthesia was maintained by additional injection of pentobarbital sodium (10 mg/kg) every 1–1.5 h. Finally, the animals were euthanized with an overdose of pentobarbital sodium (400 mg/kg) at the end of the experiments.

Electrophysiological assays of the cochleae of live guinea pigs were carried out using a procedure similar to that in our previous studies.[25-28] Double-barreled K^+-selective microelectrodes and single-barreled microelectrodes, which were prepared and calibrated as described in the above studies, were used to measure electrochemical properties of the lateral wall and EP, respectively. Although the values obtained by the K^+-selective electrodes represented K^+ activities, the ionic concentrations were calculated from these data by means of the activity coefficient for K^+ of 0.727.[25,28,29] To insert double-barreled electrodes into the cochlea, a fenestra of less than 100 μm in diameter was made on the bony wall of the second turn using a microchisel. By means of an Ag/AgCl wire on the neck muscles as a reference, a K^+-selective microelectrode was inserted into the fenestra and advanced from perilymph toward endolymph by a micromanipulator (MP-285; Sutter Instrument Co., Novato, CA, USA) to record both the potential and aK^+ of the lateral cochlear wall.

2-II. Perfusion of the perilymphatic space

To perfuse solutions into the perilymphatic space, inlet and outlet holes were created on the basal cochlear turn of the scala tympani and the third turn of the scala vestibuli, respectively. Perfusates were applied at a rate of 10 μL/min using a syringe pump through a capillary tube inserted into the inlet hole as described elsewhere.[26-28] Although native perilymph likely contains [Na^+] of 130–150 mM and [K^+] of ~5 mM, as control artificial perilymph, we used a solution with the following ionic composition (in mM); 100 Na^+, 5 K^+, 46 N-methyl D-glucose (NMDG), 1.2 Ca^{2+}, 1.0 Mg^{2+}, 131.4 Cl^-, 24 HCO_3^-, 5.0 HEPES, and 4.0 glucose saturated with 5% O_2, 5% CO_2, 90% N_2 at pH 7.4 and 295 mOsm as we reported elsewhere.[28,30] Continuous perfusion of the control solution into the scala tympani had little or no effect on EP.[28] Ouabain (10 or 50 μM) was added to this solution.

Supplementary Figure Legends

Supplementary Figure 1 Histological analysis of the lateral cochlear wall.

a shows a low-magnification image of the second turn of the cochlea of a guinea pig. The cryothin section was stained with hematoxylin and eosin and examined under a light microscope. The areas confined by *red* and *black lines* indicate the stria vascularis (StV) and the spiral ligament (SL), respectively. The regions marked by white squares in StV and SL are enlarged in ***b*** and ***c***. These data were subjected to morphometric analyses to determine the number of cells in the two tissues (see

Model development in the **main text**). SM: scala media, SV: scala vestibuli, ST: scala tympani, OC: organ of Corti.

Supplementary Figure 2 Estimation of the blocking rate of syncytial Na^+,K^+-ATPases for simulation during perilymphatic perfusion with ouabain.

(**a**) Determination of the blocking rate of syncytial Na^+,K^+-ATPase ($\kappa_{\mathrm{Ouabain,FC}}$). The simulations of the potentials and [K^+] in various compartments of the lateral wall and EP are displayed, with variation in $\kappa_{\mathrm{Ouabain,FC}}$ from 0.6 to 0.3 (see **Model development** in the **main text**). *Gray rectangles* indicate ranges of the steady-state values of the experimental measurements conducted in the cochleae of guinea pigs at 40 min after the onset of the perilymphatic perfusion with 10 μM ouabain (mean ± SD, see **Table 1**). When $\kappa_{\mathrm{Ouabain,FC}}$ was set to 0.46, EP, ISP, v_{SB}, and [K^+]$_{\mathrm{SY}}$ converged within the expected ranges.

(***b*** and ***c***) Electrochemical dynamics in the lateral-wall compartments under different conditions. The simulation was performed using the fi-NHK model under normal conditions or with blockage of syncytial Na^+,K^+-ATPases ($\kappa_{\mathrm{Ouabain,FC}}$ = 0.46; see ***a***). ***b*** shows the potentials in extracellular and intracellular spaces (*left panel*, reference: perilymph) and those across different membrane domains (*right panel*, reference: the neighboring extracellular space). In ***c***, ion concentrations in various extracellular and intracellular spaces are plotted. In the assays of panels ***a***–***c***, the activity of marginal-cell Na^+,K^+-ATPases remained unchanged, and the initial values of the potential and ion concentrations were the data obtained at 600 s after the start of the simulation.

EP: endocochlear potential, ISP: intrastrial potential, MCP: marginal-cell potential, *v*: membrane potential, MA: apical surface of marginal-cell layer, MB: basolateral surface of marginal-cell layer, SA: apical surface of syncytial layer, SB: basolateral surface of syncytial layer, HB: basolateral membrane of hair cells, PL: perilymph, IS: intrastrial space, HC: hair cells, SY: syncytial layer, MC: marginal cells, EL: endolymph.

Supplementary Figure 3 Dynamics of the current across various compartments of the lateral wall.

Panels ***a***, ***b***, and ***c*** depict respectively K^+, Na^+, and Cl^- currents flowing through the ion transport machineries in both the basolateral and apical surfaces of the syncytial and marginal-cell layer under normal and Na^+,K^+-ATPase blockage conditions. The sum of K^+ and Na^+ currents carried by all the machineries across the basolateral membrane of the syncytial layer (***a*** and ***b***, respectively) corresponds to the net K^+ and Na^+ currents described in the *upper* and *lower panels* in **Figure 4b**,

respectively; this sum is also applicable to Cl^- currents shown in ***c***. In ***a***, the behavior of the circulation current is overlaid in blue for comparison. NaKATP: Na^+,K^+-ATPases, Leak: Leak channels, Kir: Kir4.1, NSC: Nonselective cation channels, NKCC: Na^+,K^+,$2Cl^-$-cotransporters, Natrans: Na^+ transporter.

Supplementary Tables

Supplementary Table 1 Parameters used in the fi-NHK model.

Parameter	Value	References
$G_{NaKATP,FC}$	2.2×10^{-11} S/cell	Methods
$G_{NaConductance,FC}$	1.2×10^{-9} S/cell	Methods
$G_{Leak,FC}$	0.6×10^{-9} S/cell	Methods
$G_{Kir,IC}$	63.3×10^{-9} S/cell	8,23,31
$G_{Leak,IC}$	12.0×10^{-9} S/cell	1,8,23
$G_{NaKATP,MB}$	0.1×10^{-9} S/cell	1,7
$P_{NKCC,MB}$	0.1×10^{-12} mmol	1,12
$G_{ClC,MB}$	100.0×10^{-9} S/cell	10,19
$G_{NSC,MB}$	1.0×10^{-9} S/cell	1,32
$G_{Ks,MA}$	32.0×10^{-9} S/cell	13,14
$G_{NSC,MA}$	2.0×10^{-9} S/cell	14,33
G_{MET}	6.4×10^{-9} S/cell	1,16
$G_{KConductance,HB}$	40.0×10^{-9} S/cell	34,35
C_{FC}	5×10^{-11}F/cell	21,22, Supplementary Methods
C_{IC}	5×10^{-11}F/cell	2,8,23,36
C_{MB}	5×10^{-11}F/cell	19,32,33,36
C_{MA}	5×10^{-13}F/cell	19,32,33,36
C_{HB}	3×10^{-11}F/cell	37
N_{FC}*	168	Methods
N_{IC}*	16	38
N_{MC}*	40	38
N_{HC}*	3	39
V_{PL}*	350×10^{-10}L	39
V_{IS}*	21×10^{-12}L	1
V_{EL}*	350×10^{-12}L	20
V_{cell}*	350×10^{-15}L	1, 19
$\kappa_{Ouabain,FC}$	0.46	Methods

* Values in a 10-µm thick slice of the cochlea. For a list of abbreviations, see **Definitions and abbreviations**.

Supplementary Table 2 Initial and steady-state values of simulated ion concentrations and potentials in the lateral-wall compartments

**Ion concentration**	Initial value* (mM)	Steady-state (600 sec)** (mM)	Steady-state (42000 sec)** (mM)	References
$[K^+]_{PL}$	2.9	2.9	2.9	25
$[K^+]_{SY}$	100.0	97.6	97.6	28
$[K^+]_{IS}$	4.3	6.1	6.1	25
$[K^+]_{MC}$	131.0	128.2	128.2	25
$[K^+]_{EL}$	160.0	160.0	160.0	25
$[K^+]_{HC}$	133.0	133.0	133.0	SI
$[Na^+]_{PL}$	133.0	133.0	133.0	24
$[Na^+]_{SY}$	30.0	32.4	32.4	24
$[Na^+]_{IS}$	132.0	132.0	132.0	24
$[Na^+]_{MC}$	4.0	4.0	4.0	24
$[Na^+]_{EL}$	3.0	3.0	3.0	24
$[Na^+]_{HC}$	3.0	3.0	3.0	SI
$[Cl^-]_{PL}^{\#}$	106.7	106.7	106.7	1
$[Cl^-]_{SY}^{\#}$	70.0	70.0	70.0	1
$[Cl^-]_{IS}$	90.0	91.9	91.9	1
$[Cl^-]_{MC}$	112.0	109.2	109.2	1
$[Cl^-]_{EL}^{\#}$	160.0	160.0	160.0	1
$[Cl^-]_{HC}^{\#}$	70.0	70.0	70.0	SI
**Potential**	Initial value* (mV)	Steady-state (600 sec)** (mV)	Steady-state (42000 sec)** (mV)	References
v_{SB}	9.0	9.7	9.7	28
v_{SA}	-89.5	-71.4	-71.4	25
v_{MB}	-1.3	3.7	3.7	25
v_{MA}	12.6	12.1	12.1	25
v_{HB}	-76.1	-77.7	-77.7	37

Constant values. * Values initially applied to the model[1]. ** Steady-state values developed during 600 and 42000 sec. For a list of abbreviations, see **Definitions and abbreviations**. SI: Supplementary Information.

Supplementary Table 3 Effects of perilymphatic perfusion with ouabain at different concentrations

Potential or [K^+]	10 µM ouabain (n = 4)	50 µM ouabain (n = 4)	*p* value
Normal EP	+83.2 ± 3.6 mV	+88.0 ± 5.7 mV	0.5624
Changed EP*	+5.8 ± 6.2 mV	+7.5 ± 8.7 mV	0.6611
ΔEP	+81.0 ± 7.2 mV	+80.5 ± 12.0 mV	0.9509
Normal ISP	+68.7 ± 5.3 mV	+68.5 ± 10.9 mV	0.9716
Changed ISP*	+10.6 ± 4.4 mV	+14.4 ± 2.0 mV	0.1728
ΔISP	+58.1 ± 6.4 mV	+54.2 ± 12.9 mV	0.6033
Normal $[K^+]_{IS}$	8.6 ± 2.5 mM	7.5 ± 1.7 mM	0.5672
Changed $[K^+]_{IS}$*	4.2 ± 1.4 mM	4.7 ± 1.5 mM	0.9304
$\Delta[K^+]_{IS}$	-3.6 ± 1.1 mM	-2.8 ± 2.0 mM	0.7212

*Values obtained at 40 min after the onset of perfusion with ouabain.

Definitions and abbreviations.

NHK model	Nin-Hibino-Kurachi model
fi-NHK model	fibrocyte-integrated NHK model

FC	Fibrocyte
BC	Basal cell
IC	Intermediate cell
SY	Syncytial layer
MC	Marginal cell
HC	Hair cell

SM	Scala media
ST	Scala tympani
SV	Scala vestibuli
PL	Perilymph
IS	Intrastrial space
EL	Endolymph

SB	Basolateral surface of syncytial layer
SA	Apical surface of syncytial layer
MB	Basolateral surface of marginal-cell layer
MA	Apical surface of marginal-cell layer
HA	Apical membrane of hair cell
HB	Basolateral membrane of hair cell

RMP	Resting membrane potential
EP	Endocochlear potential
ISP	Intrastrial potential
MCP	Marginal-cell potential
v	Membrane potential
v_{SB}	Membrane potential of basolateral surface of syncytial layer, mV
v_{SA}	Membrane potential of apical surface of syncytial layer, mV

v_{MB}	Membrane potential in basolateral membrane of marginal cell, mV
v_{MA}	Membrane potential in apical membrane of marginal cell, mV
v_{HA}	Membrane potential in apical membrane of hair cell, mV
v_{HB}	Membrane potential in basolateral membrane of hair cell, mV

C_{FC}	Capacitance of fibrocyte membrane, F/cell
C_{SB}	Capacitance of basolateral surface of syncytial layer, F
C_{IC}	Capacitance of intermediate cell membrane, F/cell
C_{SA}	Capacitance of apical surface of syncytial layer, F
C_{MB}	Capacitance in basolateral membrane of marginal cell, F/cell
C_{MA}	Capacitance in apical membrane of marginal cell, F/cell
C_{HB}	Capacitance in basolateral membrane of hair cell, F/cell

N_{FC}	Number of fibrocytes
N_{IC}	Number of intermediate cells
N_{MC}	Number of marginal cells
N_{HC}	Number of hair cells

V_{PL}	Volume of fibrocyte, L/section
V_{IS}	Volume of intrastrial space, L/section
V_{EL}	Volume of endolymph, L/section
V_{Cell}	Volume of cell (Fibrocyte, Intermediate cell, Marginal cell, Hair cell), L/section

$[K^+]_{\mathrm{PL}}$	K^+ concentration in perilymph, mmol/L
$[K^+]_{\mathrm{SY}}$	K^+ concentration in syncytium, mmol/L
$[K^+]_{\mathrm{IS}}$	K^+ concentration in intrastrial space, mmol/L
$[K^+]_{\mathrm{MC}}$	K^+ concentration in marginal cell, mmol/L
$[K^+]_{\mathrm{EL}}$	K^+ concentration in endolymph, mmol/L
$[K^+]_{\mathrm{HC}}$	K^+ concentration in hair cell, mmol/L
$[Na^+]_{\mathrm{PL}}$	Na^+ concentration in perilymph, mmol/L
$[Na^+]_{\mathrm{SY}}$	Na^+ concentration in syncytium, mmol/L
$[Na^+]_{\mathrm{IS}}$	Na^+ concentration in intrastrial space, mmol/L
$[Na^+]_{\mathrm{MC}}$	Na^+ concentration in marginal cell, mmol/L

$[Na^+]_{EL}$ — Na^+ concentration in endolymph, mmol/L
$[Na^+]_{HC}$ — Na^+ concentration in hair cell, mmol/L
$[Cl^-]_{PL}$ — Cl^- concentration in perilymph, mmol/L
$[Cl^-]_{SY}$ — Cl^- concentration in syncytium, mmol/L
$[Cl^-]_{IS}$ — Cl^- concentration in intrastrial space, mmol/L
$[Cl^-]_{MC}$ — Cl^- concentration in marginal cell, mmol/L
$[Cl^-]_{EL}$ — Cl^- concentration in endolymph, mmol/L
$[Cl^-]_{HC}$ — Cl^- concentration in hair cell, mmol/L

NaKATP — Na^+,K^+-ATPase
NKCC — Na^+,K^+,2Cl^- cotransporter
Kir4.1 — Kir4.1 K^+ conductance
ClC — ClC/K Cl^- conductance
KCNQ1/KCNE1 — KCNQ1/KCNE1 K^+ conductance
Leak — Leak conductance
NSC — Nonselective cation conductance
Na^+-T — Na^+ transporter

I_{FC} — Current on fibrocyte, A/cell
I_{SB} — Current on basolateral surface of syncytial layer, A
$I_{Na,FC}$ — Na^+ current through membrane of fibrocyte, A/cell
$I_{K,FC}$ — K^+ current through membrane of fibrocyte, A/cell
$I_{NaKATP,FC}$ — Flow via Na^+,K^+-ATPases on membrane of fibrocyte, A/cell
$f_{NaKATP,FC}$ — Voltage-dependent parameter of $I_{NaKATP,FC}$
σ_{FC} — $[Na^+]$-dependent factor of $f_{NaKATP,FC}$
$I_{K,NaKATP,FC}$ — K^+ component of $I_{NaKATP,FC}$, A/cell
$I_{Na,NaKATP,FC}$ — Na^+ component of $I_{NaKATP,FC}$, A/cell
$I_{NaConductance,FC}$ — Current through Na^+ conductance on membrane of fibrocyte, A/cell
$I_{Leak,FC}$ — Current through leak conductance on membrane of fibrocyte, A/cell
$I_{K,Leak,FC}$ — K^+ component of $I_{Leak,FC}$, A/cell
$I_{Na,Leak,FC}$ — Na^+ component of $I_{Leak,FC}$, A/cell
$G_{NaKATP,FC}$ — Activity of Na^+,K^+-ATPase on a fibrocyte membrane, S/cell
$G_{NaConductance,FC}$ — Na^+ conductance on fibrocyte membrane, S/cell

$G_{Leak,FC}$	Leak conductance on fibrocyte membrane, S/cell
I_{IC}	Current on intermediate cell, A/cell
I_{SA}	Current on apical surface of syncytial layer, A
$I_{Na,IC}$	Na^+ current on membrane of intermediate cell, A/cell
$I_{K,IC}$	K^+ current on membrane of intermediate cell, A/cell
$I_{Kir,IC}$	K^+ current through Kir4.1 K^+ conductance on membrane of intermediate cell, A/cell
$I_{Leak,IC}$	Current through leak conductance on membrane of intermediate cell, A/cell
$I_{K,Leak,IC}$	K^+ component of $I_{Leak,IC}$, A/cell
$I_{Na,Leak,IC}$	Na^+ component of $I_{Leak,IC}$, A/cell
$I_{Na,trans,IC}$	Flow through Na^+ transporter in intermediate cell, A/cell
$G_{Kir,IC}$	Conductance of Kir 4.1 K^+ conductance on intermediate cell, S/cell
$G_{Leak,IC}$	Leak conductance on intermediate cell, S/cell
I_{MB}	Current on basolateral membrane of marginal cell, A/cell
$I_{NaKATP,MB}$	Flow via Na^+,K^+-ATPase on basolateral membrane of marginal cell, A/cell
$f_{NaKATP,MB}$	Voltage-dependent parameter of $I_{NaKATP, MB}$
σ_{MB}	[Na^+]-dependent factor of $f_{NaKATP, MB}$
$I_{K,NaKATP,MB}$	K^+ component of $I_{NaKATP,MB}$, A/cell
$I_{Na,NaKATP,MB}$	Na^+ component of $I_{NaKATP,MB}$, A/cell
$I_{NKCC,MB}$	Flow via NKCCs on basolateral membrane of marginal cell, A/cell
k,α,β	rate constants, s^{-1}
$P_{NKCC,MB}$	Factor to define the flux via NKCCs on basolateral membrane of marginal cell, mmol
$p(X)$	probability of state X in multiple-state gate
$I_{K,NKCC,MB}$	K^+ component of $I_{NKCC,MB}$, A/cell
$I_{Na,NKCC,MB}$	Na^+ component of $I_{NKCC,MB}$, A/cell
$I_{Cl,NKCC,MB}$	Cl^- component of $I_{NKCC,MB}$, A/cell
$I_{ClC,MB}$	Cl^- current through ClC/K-barttin Cl^- conductance in basolateral membrane of marginal cell, A/cell
$I_{NSC,MB}$	Current through NSC conductance on basolateral membrane of marginal cell, A/cell

$I_{\mathrm{K,NSC,MB}}$	K^+ component of $I_{\mathrm{NSC,MB}}$, A/cell
$I_{\mathrm{Na,NSC,MB}}$	Na^+ component of $I_{\mathrm{NSC,MB}}$, A/cell
$G_{\mathrm{NaKATP,MB}}$	Activity of Na^+,K^+-ATPase on basolateral membrane of marginal cell, S/cell
$P_{\mathrm{NKCC,MB}}$	Factor to define flux via NKCCs on basolateral membrane of marginal cell, S/cell
K_{Na}	Na^+-binding constant of NKCC, L/mol
K_{K}	K^+-binding constant of NKCC, L/mol
K_{Cl}	Cl^--binding constant of NKCC, L/mol
k^f_{full}	Rate constant of ion-filled NKCC, /s
k^b_{full}	Rate constant of ion-filled NKCC, /s
k^f_{empty}	Rate constant of NKCC in empty form, /s
k^b_{empty}	Rate constant of NKCC in empty form, /s
$G_{\mathrm{ClC,MB}}$	Conductance of ClC/K-barttin Cl^- conductance on basolateral membrane of marginal cell, S/cell
$G_{\mathrm{NSC,MB}}$	Conductance of NSC conductance on basolateral membrane of marginal cell, S/cell
I_{MA}	Current on apical membrane of marginal cell, A/cell
$I_{\mathrm{Ks,MA}}$	K^+ current through KCNQ1/KCNE1 K^+ conductance channels on apical membrane of marginal cell, A/cell
n	The open probability of activation gate
n_{∞}	The steady state open probability of activation gate n
τ_{Ks}	Time constant of activation of KCNQ1/KCNE1 K^+ conductance
$I_{\mathrm{NSC,MA}}$	Current through NSC conductance in apical membrane of marginal cell, A/cell
$I_{\mathrm{K,NSC,MA}}$	K^+ component of $I_{\mathrm{NSC,MA}}$, A/cell
$I_{\mathrm{Na,NSC,MA}}$	Na^+ component of $I_{\mathrm{NSC,MA}}$, A/cell
$G_{\mathrm{Ks,MA}}$	Conductance of KCNQ1/KCNE1 K^+ conductance on apical membrane of marginal cell, S/cell
$G_{\mathrm{NSC,MA}}$	Conductance of NSC conductance on apical membrane of marginal cell, S/cell
I_{MET}	Current of MET channels on apical membrane of hair cell, A/cell
G_{MET}	Conductance of MET channels on apical membrane of hair cell, A/cell
I_{Cir}	Circulation current, A

I_{HB}	Current on basolateral membrane of marginal cell, A/cell
$I_{KConductance,HB}$	Current through K^+ conductance on basolateral membrane of hair cell, A/cell
$G_{KConductance,HB}$	Conductance of Ca^{2+}-activated K^+ conductance on basolateral membrane of hair cell, S/cell
$\kappa_{Ouabain,FC}$	Factor of blocking rate of Na^+,K^+-ATPase in fibrocyte during ouabain perilymphatic perfusion
F	Faraday constant, 96,487 C/mol
R	Gas constant, 8314.47 J/kmol/K
T	Temperature, 310.15 kelvin

Formulation of the model.

I. Currents and capacitances in the syncytial layer.

$I_{\mathrm{SB}} = N_{\mathrm{FC}} \cdot I_{\mathrm{FC}}$

$I_{\mathrm{SA}} = N_{\mathrm{IC}} \cdot I_{\mathrm{IC}}$

$C_{\mathrm{SB}} = N_{\mathrm{FC}} \cdot C_{\mathrm{FC}}$

$C_{\mathrm{SA}} = N_{\mathrm{IC}} \cdot C_{\mathrm{IC}}$

II. Membrane potentials.

$$\frac{dv_{\mathrm{SB}}}{dt} = \frac{-I_{\mathrm{SB}}-I_{\mathrm{Cir}}}{C_{\mathrm{SB}}} = \frac{-N_{\mathrm{FC}} \cdot I_{\mathrm{FC}}-I_{\mathrm{Cir}}}{N_{\mathrm{FC}} \cdot C_{\mathrm{FC}}} = \frac{N_{\mathrm{HC}} \cdot I_{\mathrm{MET}}-N_{\mathrm{FC}} \cdot \left(I_{\mathrm{NaKATP,FC}}+I_{\mathrm{NaConductance,FC}}+I_{\mathrm{Leak,FC}}\right)}{N_{\mathrm{FC}} \cdot C_{\mathrm{FC}}}$$

$$\frac{dv_{\mathrm{SA}}}{dt} = \frac{I_{\mathrm{Cir}}-I_{\mathrm{SA}}}{C_{\mathrm{SA}}} = \frac{I_{\mathrm{Cir}}-N_{\mathrm{IC}} \cdot I_{\mathrm{IC}}}{N_{\mathrm{IC}} \cdot C_{\mathrm{IC}}} = \frac{-N_{\mathrm{HC}} \cdot I_{\mathrm{MET}}-N_{\mathrm{IC}} \cdot \left(I_{\mathrm{Kir,IC}}+I_{\mathrm{Leak,IC}}+I_{\mathrm{Na,trans,IC}}\right)}{N_{\mathrm{IC}} \cdot C_{\mathrm{IC}}}$$

$$\frac{dv_{\mathrm{MB}}}{dt} = \frac{-N_{\mathrm{MC}} \cdot I_{\mathrm{MB}}-I_{\mathrm{Cir}}}{N_{\mathrm{MC}} \cdot C_{\mathrm{MB}}} = \frac{N_{\mathrm{HC}} \cdot I_{\mathrm{MET}}-N_{\mathrm{MC}} \cdot \left(I_{\mathrm{ClC,MB}}+I_{\mathrm{NSC,MB}}+I_{\mathrm{NaKATP,MB}}\right)}{N_{\mathrm{MC}} \cdot C_{\mathrm{MB}}}$$

$$\frac{dv_{\mathrm{MA}}}{dt} = \frac{I_{\mathrm{Cir}}-N_{\mathrm{MC}} \cdot I_{\mathrm{MA}}}{N_{\mathrm{MC}} \cdot C_{\mathrm{MA}}} = \frac{-N_{\mathrm{HC}} \cdot I_{\mathrm{MET}}-N_{\mathrm{MC}} \cdot \left(I_{\mathrm{Ks,MA}}+I_{\mathrm{NSC,MA}}+I_{\mathrm{Na,trans,MA}}\right)}{N_{\mathrm{MC}} \cdot C_{\mathrm{MA}}}$$

$$\frac{dv_{\mathrm{HB}}}{dt} = \frac{I_{\mathrm{Cir}}-N_{\mathrm{HC}} \cdot I_{\mathrm{HB}}}{N_{\mathrm{HC}} \cdot C_{\mathrm{HB}}} = \frac{-N_{\mathrm{HC}} \cdot I_{\mathrm{MET}}-N_{\mathrm{HC}} \cdot I_{\mathrm{KConductance,HB}}}{N_{\mathrm{HC}} \cdot C_{\mathrm{HB}}}$$

III. Ionic concentrations in intracellular and extracellular fluids.

$$\frac{d[\mathrm{K}^+]_{\mathrm{PL}}}{dt} = \frac{N_{\mathrm{HC}} \cdot I_{\mathrm{HB}}+N_{\mathrm{FC}} \cdot \left(I_{\mathrm{K,NaKATP,FC}}+I_{\mathrm{K,Leak,FC}}\right)}{V_{\mathrm{PL}} \cdot F}$$

$$\frac{d[\mathrm{K}^+]_{\mathrm{SY}}}{dt} = -\frac{N_{\mathrm{FC}} \cdot \left(I_{\mathrm{K,NaKATP,FC}}+I_{\mathrm{K,Leak,FC}}\right)+N_{\mathrm{IC}} \cdot \left(I_{\mathrm{Kir,IC}}+I_{\mathrm{K,Leak,IC}}\right)}{\left(N_{\mathrm{FC}}+N_{\mathrm{IC}}\right) \cdot V_{\mathrm{Cell}} \cdot F}$$

$$\frac{d[\mathrm{K}^+]_{\mathrm{IS}}}{dt} = \frac{N_{\mathrm{IC}} \cdot \left(I_{\mathrm{Kir,IC}}+I_{\mathrm{K,Leak,IC}}\right)+N_{\mathrm{MC}} \cdot \left(I_{\mathrm{K,NSC,MB}}+I_{\mathrm{K,NaKATP,MB}}+I_{\mathrm{K,NKCC,MB}}\right)}{V_{\mathrm{IS}} \cdot F}$$

$$\frac{d[\mathrm{K}^+]_{\mathrm{MC}}}{dt} = -\frac{N_{\mathrm{MC}} \cdot \left(I_{\mathrm{K,NSC,MB}}+I_{\mathrm{K,NaKATP,MB}}+I_{\mathrm{K,NKCC,MB}}\right)+N_{\mathrm{MC}} \cdot \left(I_{\mathrm{Ks,MA}}+I_{\mathrm{K,NSC,MA}}\right)}{N_{\mathrm{MC}} \cdot V_{\mathrm{Cell}} \cdot F}$$

$$\frac{d[\mathrm{K}^+]_{\mathrm{EL}}}{dt} = \frac{N_{\mathrm{MC}} \cdot \left(I_{\mathrm{Ks,MA}}+I_{\mathrm{K,NSC,MA}}\right)+N_{\mathrm{HC}} \cdot I_{\mathrm{MET}}}{V_{\mathrm{EL}} \cdot F}$$

$$\frac{d[\mathrm{K}^+]_{\mathrm{HC}}}{dt} = -\frac{N_{\mathrm{HC}} \cdot I_{\mathrm{MET}}+N_{\mathrm{HC}} \cdot I_{\mathrm{KConductance,HB}}}{N_{\mathrm{HC}} \cdot V_{\mathrm{Cell}} \cdot F}$$

$$\frac{d[\mathrm{Na}^{+}]_{\mathrm{PL}}}{dt}=\frac{N_{\mathrm{FC}}\cdot\left(I_{\mathrm{Na,NaKATP,FC}}+I_{\mathrm{NaConductance,FC}}+I_{\mathrm{Na,Leak,FC}}\right)}{V_{\mathrm{PL}}\cdot F}$$

$$\frac{d[\mathrm{Na}^{+}]_{\mathrm{SY}}}{dt}=-\frac{N_{\mathrm{FC}}\cdot\left(I_{\mathrm{Na,NaKATP,FC}}+I_{\mathrm{NaConductance,FC}}+I_{\mathrm{Na,Leak,FC}}\right)+N_{\mathrm{IC}}\cdot\left(I_{\mathrm{Na,Leak,IC}}+I_{\mathrm{Na,trans,IC}}\right)}{(N_{\mathrm{FC}}+N_{\mathrm{IC}})\cdot V_{\mathrm{Cell}}\cdot F}$$

$$\frac{d[\mathrm{Na}^{+}]_{\mathrm{IS}}}{dt}=\frac{N_{\mathrm{IC}}\cdot\left(I_{\mathrm{Na,Leak,FC}}+I_{\mathrm{Na,trans,FC}}\right)+N_{\mathrm{MC}}\cdot\left(I_{\mathrm{Na,NSC,MB}}+I_{\mathrm{Na,NaKATP,MB}}+I_{\mathrm{Na,NKCC,MB}}\right)}{V_{\mathrm{IS}}\cdot F}$$

$$\frac{d[\mathrm{Na}^{+}]_{\mathrm{MC}}}{dt}=-\frac{N_{\mathrm{MC}}\cdot\left(I_{\mathrm{Na,NSC,MB}}+I_{\mathrm{Na,NaKATP,MB}}+I_{\mathrm{Na,NKCC,MB}}\right)+N_{\mathrm{MC}}\cdot\left(I_{\mathrm{Na,NSC,MA}}+I_{\mathrm{Na,trans,MA}}\right)}{N_{\mathrm{MC}}\cdot V_{\mathrm{Cell}}\cdot F}$$

$$\frac{d[\mathrm{Na}^{+}]_{\mathrm{EL}}}{dt}=\frac{N_{\mathrm{MC}}\cdot\left(I_{\mathrm{Na,NSC,MA}}+I_{\mathrm{Na,trans,MA}}\right)}{V_{\mathrm{EL}}\cdot F}$$

$$\frac{d[\mathrm{Na}^{+}]_{\mathrm{HC}}}{dt}=0$$

$$\frac{d[\mathrm{Cl}^{-}]_{\mathrm{PL}}}{dt}=0$$

$$\frac{d[\mathrm{Cl}^{-}]_{\mathrm{SY}}}{dt}=0$$

$$\frac{d[\mathrm{Cl}^{-}]_{\mathrm{IS}}}{dt}=-\frac{N_{\mathrm{MC}}\cdot\left(I_{\mathrm{ClC,MB}}+I_{\mathrm{Cl,NKCC,MB}}\right)}{V_{\mathrm{IS}}\cdot F}$$

$$\frac{d[\mathrm{Cl}^{-}]_{\mathrm{MC}}}{dt}=\frac{N_{\mathrm{MC}}\cdot\left(I_{\mathrm{ClC,MB}}+I_{\mathrm{Cl,NKCC,MB}}\right)}{N_{\mathrm{MC}}\cdot V_{\mathrm{Cell}}\cdot F}$$

$$\frac{d[\mathrm{Cl}^{-}]_{\mathrm{EL}}}{dt}=0$$

$$\frac{d[\mathrm{Cl}^{-}]_{\mathrm{HC}}}{dt}=0$$

IV. Ionic flows and currents.

a. MET current and circulation current: I_{MET} and I_{Cir}

$$I_{\mathrm{MET}}=G_{\mathrm{MET}}\left(v_{\mathrm{HA}}-\frac{RT}{F}\cdot ln\left(\frac{[\mathrm{K}^{+}]_{\mathrm{EL}}}{[\mathrm{K}^{+}]_{\mathrm{HC}}}\right)\right)$$

$$I_{\mathrm{Cir}}=-N_{\mathrm{HC}}\cdot I_{\mathrm{MET}}$$

b. Current through Ca^{2+}-activated K^{+} conductance on a basolateral membrane of a hair cell: $I_{\mathrm{KConductance,HB}}$

$$I_{\text{KConductance,HB}} = G_{\text{KConductance,HB}} \cdot \left(v_{\text{HB}} - \frac{RT}{F} \cdot ln\left(\frac{[\text{K}^+]_{\text{PL}}}{[\text{K}^+]_{\text{HC}}}\right)\right)$$

c. Current through Na$^+$ conductance in a fibrocyte: $I_{\text{NaConductance,FC}}$

$$I_{\text{NaConductance,FC}} = G_{\text{NaConductance,FC}} \cdot \left(v_{\text{SB}} - \frac{RT}{F} \cdot ln\left(\frac{[\text{Na}^+]_{\text{PL}}}{[\text{Na}^+]_{\text{SY}}}\right)\right)$$

d. Current through leak conductance in a fibrocyte: $I_{\text{Leak,FC}}$

$$I_{\text{Leak,FC}} = G_{\text{Leak,FC}} \cdot \left(v_{\text{SB}} - \frac{RT}{F} \cdot ln\left(\frac{[\text{K}^+]_{\text{PL}}+[\text{Na}^+]_{\text{PL}}}{[\text{K}^+]_{\text{SY}}+[\text{Na}^+]_{\text{SY}}}\right)\right)$$

$$If\ I_{\text{Leak,FC}} > 0,$$

$$I_{\text{K,Leak,FC}} = \left(\frac{[\text{K}^+]_{\text{SY}}}{[\text{K}^+]_{\text{SY}}+[\text{Na}^+]_{\text{SY}}}\right) \cdot I_{\text{Leak,FC}}$$

$$I_{\text{Na,Leak,FC}} = \left(\frac{[\text{Na}^+]_{\text{SY}}}{[\text{K}^+]_{\text{SY}}+[\text{Na}^+]_{\text{SY}}}\right) \cdot I_{\text{Leak,FC}}$$

$$If\ I_{\text{Leak,FC}} \leq 0,$$

$$I_{\text{K,Leak,FC}} = \left(\frac{[\text{K}^+]_{\text{PL}}}{[\text{K}^+]_{\text{PL}}+[\text{Na}^+]_{\text{PL}}}\right) \cdot I_{\text{Leak,FC}}$$

$$I_{\text{Na,Leak,FC}} = \left(\frac{[\text{Na}^+]_{\text{PL}}}{[\text{K}^+]_{\text{PL}}+[\text{Na}^+]_{\text{PL}}}\right) \cdot I_{\text{Leak,FC}}$$

e. Flow via Na$^+$,K$^+$-ATPase in a fibrocyte: $I_{\text{NaKATP,FC}}$

$$I_{\text{NaKATP,FC}} = \kappa_{\text{Ouabain,FC}} \cdot G_{\text{NaKATP,FC}} \cdot \frac{f_{\text{NaKATP,FC}}}{1+\left(\frac{10}{[\text{Na}^+]_{\text{SY}}}\right)^{1.5}} \cdot \frac{1}{1+\frac{1.5}{[\text{K}^+]_{\text{PL}}}}$$

$$f_{\text{NaKATP,FC}} = \frac{1}{1+0.1245 \cdot e^{\frac{-0.1 \cdot v_{\text{SB}} \cdot F}{RT}} + 0.0365 \cdot \sigma_{\text{FC}} \cdot e^{\frac{-v_{\text{SB}} \cdot F}{RT}}}$$

$$\sigma_{\text{FC}} = \frac{e^{\frac{[\text{Na}^+]_{\text{PL}}}{67.3}} - 1}{7}$$

f. Current through Kir4.1 K$^+$ conductance in an intermediate cell: $I_{\text{Kir,IC}}$

$$I_{\text{Kir,IC}} = G_{\text{Kir,IC}} \cdot \sqrt{[\text{K}^+]_{\text{IS}}} \cdot \left(\frac{v_{\text{SA}} - \frac{RT}{F} \cdot ln\left(\frac{[\text{K}^+]_{\text{IS}}}{[\text{K}^+]_{\text{SY}}}\right)}{1+e^{\frac{v_{\text{SA}}+0.05928}{0.04846}}}\right)$$

g. Current through leak conductance in an intermediate cell: $I_{\mathrm{Leak,IC}}$

$$I_{\mathrm{Leak,IC}} = G_{\mathrm{Leak,IC}} \cdot \left(v_{\mathrm{SA}} - \frac{RT}{F} \cdot ln\left(\frac{[\mathrm{K^+}]_{\mathrm{IS}} + [\mathrm{Na^+}]_{\mathrm{IS}}}{[\mathrm{K^+}]_{\mathrm{SY}} + [\mathrm{Na^+}]_{\mathrm{SY}}} \right) \right)$$

$If\ I_{\mathrm{Leak,IC}} > 0,$

$$I_{\mathrm{K,Leak,IC}} = \left(\frac{[\mathrm{K^+}]_{\mathrm{SY}}}{[\mathrm{K^+}]_{\mathrm{SY}} + [\mathrm{Na^+}]_{\mathrm{SY}}} \right) \cdot I_{\mathrm{Leak,IC}}$$

$$I_{\mathrm{Na,Leak,IC}} = \left(\frac{[\mathrm{Na^+}]_{\mathrm{SY}}}{[\mathrm{K^+}]_{\mathrm{SY}} + [\mathrm{Na^+}]_{\mathrm{SY}}} \right) \cdot I_{\mathrm{Leak,IC}}$$

$If\ I_{Leak,IC} \leq 0,$

$$I_{\mathrm{K,Leak,IC}} = \left(\frac{[\mathrm{K^+}]_{\mathrm{IS}}}{[\mathrm{K^+}]_{\mathrm{IS}} + [\mathrm{Na^+}]_{\mathrm{IS}}} \right) \cdot I_{\mathrm{Leak,IC}}$$

$$I_{\mathrm{Na,Leak,IC}} = \left(\frac{[\mathrm{Na^+}]_{\mathrm{IS}}}{[\mathrm{K^+}]_{\mathrm{IS}} + [\mathrm{Na^+}]_{\mathrm{IS}}} \right) \cdot I_{\mathrm{Leak,IC}}$$

h. Flow via $\mathrm{Na^+}$ transporter in an intermediate cell: $I_{\mathrm{Na,trans,IC}}$

$$I_{\mathrm{Na,trans,IC}} = -I_{\mathrm{Na,NSC,IC}}$$

i. Flow via $\mathrm{Na^+}$,$\mathrm{K^+}$-ATPases on a basolateral membrane of a marginal cell: $I_{\mathrm{NaKATP,MB}}$

$$I_{\mathrm{NaKATP,MB}} = G_{\mathrm{NaKATP,MB}} \cdot \frac{f_{\mathrm{NaKATP,MB}}}{1 + \left(\frac{10}{[\mathrm{Na^+}]_{\mathrm{MC}}} \right)^{1.5}} \cdot \frac{1}{1 + \frac{1.5}{[\mathrm{K^+}]_{\mathrm{IS}}}}$$

$$f_{\mathrm{NaKATP,MB}} = \frac{1}{1 + 0.1245 \cdot e^{\frac{-0.1 \cdot v_{\mathrm{MB}} \cdot F}{RT}} + 0.0365 \cdot \sigma_{\mathrm{MB}} \cdot e^{\frac{-v_{\mathrm{MB}} \cdot F}{RT}}}$$

$$\sigma_{\mathrm{MB}} = \frac{e^{\frac{[\mathrm{Na^+}]_{\mathrm{IS}}}{67.3}} - 1}{7}$$

j. Flow via $\mathrm{Na^+}$,$\mathrm{K^+}$,$\mathrm{2Cl^-}$ cotransporters in a marginal cell: $J_{\mathrm{NKCC,MB}}$

$$J_{\mathrm{NKCC,MB}} = P_{\mathrm{NKCC,MB}} \cdot \left(p\left(E_{\mathrm{1NaClKCl,MB}}\right) \cdot y \cdot k_{full}^{f} - p\left(E_{\mathrm{2NaClKCl,MB}}\right) \cdot (1 - y) \cdot k_{full}^{b} \right)$$

$$p\left(E_{1,MB}\right) =$$

$$\frac{1}{(1 + K_{Na}[\mathrm{Na^+}]_{\mathrm{IS}} + K_{Na}[\mathrm{Na^+}]_{\mathrm{IS}} K_{Cl}[\mathrm{Cl^-}]_{\mathrm{IS}} + K_{Na}[\mathrm{Na^+}]_{\mathrm{IS}} K_{Cl}[\mathrm{Cl^-}]_{\mathrm{IS}} K_{K} \cdot [\mathrm{K^+}]_{\mathrm{IS}} + K_{Na}[\mathrm{Na^+}]_{\mathrm{IS}} \cdot K_{Cl}[\mathrm{Cl^-}]_{\mathrm{IS}} K_{K}[\mathrm{K^+}]_{\mathrm{IS}} \cdot K_{Cl}[\mathrm{Cl^-}]_{\mathrm{IS}})}$$

$$p\left(E_{1NaClKCl,MB}\right) = K_{Na}[\mathrm{Na^+}]_{\mathrm{IS}} K_{Cl}[\mathrm{Cl^-}]_{\mathrm{IS}} K_{K}[\mathrm{K^+}]_{\mathrm{IS}} K_{Cl}[\mathrm{Cl^-}]_{\mathrm{IS}} \cdot p\left(E_{1,MB}\right)$$

$$p\left(E_{2,MB}\right) =$$

$$\frac{1}{(1+K_{Cl}[\mathrm{Cl}^-]_{\mathrm{MC}}+K_K[\mathrm{K}^+]_{\mathrm{MC}}K_{Cl}[\mathrm{Cl}^-]_{\mathrm{MC}}+K_K[\mathrm{K}^+]_{\mathrm{MC}}K_{Cl}[\mathrm{Cl}^-]_{\mathrm{MC}}K_K[\mathrm{K}^+]_{\mathrm{MC}}+K_{Na}[\mathrm{Na}^+]_{\mathrm{MC}}K_{Cl}[\mathrm{Cl}^-]_{\mathrm{MC}}K_K[\mathrm{K}^+]_{\mathrm{MC}}K_{Cl}[\mathrm{Cl}^-]_{\mathrm{MC}})}$$

$$p\left(E_{2NaClKCl,MB}\right) = K_{Na}[\mathrm{Na}^+]_{\mathrm{MC}}K_{Cl}[\mathrm{Cl}^-]_{\mathrm{MC}}K_K[\mathrm{K}^+]_{\mathrm{MC}}K_{Cl}[\mathrm{Cl}^-]_{\mathrm{MC}} \cdot p\left(E_{2,MB}\right)$$

$$\alpha_{MB} = k_{full}^{f} \cdot p\left(E_{1NaClKCl,MB}\right) + k_{empty}^{b} \cdot p\left(E_{1,MB}\right)$$

$$\beta_{MB} = k_{full}^{b} \cdot p\left(E_{2NaClKCl,MB}\right) + k_{empty}^{f} \cdot p\left(E_{2,MB}\right)$$

$$\frac{dy}{dt} = \beta_{MB}(1-y) - \alpha_{MB}y$$

k. Current through ClC/K-barttin Cl^- conductance on the basolateral membrane of a marginal cell: $I_{\mathrm{ClC,MB}}$

$$I_{\mathrm{ClC,MB}} = G_{\mathrm{ClC,MB}} \cdot \left(v_{\mathrm{MB}} - \frac{RT}{F} \cdot ln\left(\frac{[\mathrm{Cl}^-]_{\mathrm{MC}}}{[\mathrm{Cl}^-]_{\mathrm{IS}}}\right)\right)$$

l. Current through NSC conductance on the basolateral membrane of a marginal cell: $I_{\mathrm{NSC,MB}}$

$$I_{\mathrm{NSC,MB}} = G_{\mathrm{NSC,MB}} \cdot \left(v_{\mathrm{MB}} - \frac{RT}{F} \cdot ln\left(\frac{[\mathrm{K}^+]_{\mathrm{IS}}+[\mathrm{Na}^+]_{\mathrm{IS}}}{[\mathrm{K}^+]_{\mathrm{MC}}+[\mathrm{Na}^+]_{\mathrm{MC}}}\right)\right)$$

$$If\ I_{\mathrm{NSC,MB}} > 0,$$

$$I_{\mathrm{K,NSC,MB}} = \left(\frac{[\mathrm{K}^+]_{\mathrm{MC}}}{[\mathrm{K}^+]_{\mathrm{MC}}+[\mathrm{Na}^+]_{\mathrm{MC}}}\right) \cdot I_{\mathrm{NSC,MB}}$$

$$I_{\mathrm{Na,NSC,MB}} = \left(\frac{[\mathrm{Na}^+]_{\mathrm{MC}}}{[\mathrm{K}^+]_{\mathrm{MC}}+[\mathrm{Na}^+]_{\mathrm{MC}}}\right) \cdot I_{\mathrm{NSC,MB}}$$

$$If\ I_{\mathrm{NSC,MB}} \leq 0,$$

$$I_{\mathrm{K,NSC,MB}} = \left(\frac{[\mathrm{K}^+]_{\mathrm{IS}}}{[\mathrm{K}^+]_{\mathrm{IS}}+[\mathrm{Na}^+]_{\mathrm{IS}}}\right) \cdot I_{\mathrm{NSC,MB}}$$

$$I_{\mathrm{Na,NSC,MB}} = \left(\frac{[\mathrm{Na}^+]_{\mathrm{IS}}}{[\mathrm{K}^+]_{\mathrm{IS}}+[\mathrm{Na}^+]_{\mathrm{IS}}}\right) \cdot I_{\mathrm{NSC,MB}}$$

m. Current through KCNQ1/KCNE1 K^+ conductance on the apical membrane of a marginal cell: $I_{\mathrm{Ks,MA}}$

$$I_{\mathrm{Ks,MA}} = G_{\mathrm{Ks,MA}} \cdot n \cdot \left(v_{\mathrm{MA}} - \frac{RT}{F} \cdot ln\left(\frac{[\mathrm{K}^+]_{\mathrm{EL}}}{[\mathrm{K}^+]_{\mathrm{MC}}}\right)\right)$$

$$n_{\infty} = \left(\frac{1}{1+e^{\frac{v_{\mathrm{MA}}-0.02048}{-0.0106}}}\right)$$

$$\tau_{Ks} = 0.401 + 0.687 \cdot \left(\frac{1}{1+e^{\frac{v_{\mathrm{MA}}-0.02616}{0.00838}}}\right)$$

n. Current through NSC conductance on the apical membrane of a marginal cell: $I_{\mathrm{NSC,MA}}$

$$I_{\mathrm{NSC,MA}} = G_{\mathrm{NSC,MA}} \cdot \left(v_{\mathrm{MA}} - \frac{RT}{F} \cdot ln\left(\frac{[\mathrm{K}^{+}]_{\mathrm{EL}}+[\mathrm{Na}^{+}]_{\mathrm{EL}}}{[\mathrm{K}^{+}]_{\mathrm{MC}}+[\mathrm{Na}^{+}]_{\mathrm{MC}}}\right)\right)$$

$$If\ I_{\mathrm{NSC,MA}} > 0,$$

$$I_{\mathrm{K,NSC,MA}} = \left(\frac{[\mathrm{K}^{+}]_{\mathrm{MC}}}{[\mathrm{K}^{+}]_{\mathrm{MC}}+[\mathrm{Na}^{+}]_{\mathrm{MC}}}\right) \cdot I_{\mathrm{NSC,MA}}$$

$$I_{\mathrm{Na,NSC,MA}} = \left(\frac{[\mathrm{Na}^{+}]_{\mathrm{MC}}}{[\mathrm{K}^{+}]_{\mathrm{MC}}+[\mathrm{Na}^{+}]_{\mathrm{MC}}}\right) \cdot I_{\mathrm{NSC,MA}}$$

$$If\ I_{\mathrm{NSC,MA}} \leq 0,$$

$$I_{\mathrm{K,NSC,MA}} = \left(\frac{[\mathrm{K}^{+}]_{\mathrm{EL}}}{[\mathrm{K}^{+}]_{\mathrm{EL}}+[\mathrm{Na}^{+}]_{\mathrm{EL}}}\right) \cdot I_{\mathrm{NSC,MA}}$$

$$I_{\mathrm{Na,NSC,MA}} = \left(\frac{[\mathrm{Na}^{+}]_{\mathrm{EL}}}{[\mathrm{K}^{+}]_{\mathrm{EL}}+[\mathrm{Na}^{+}]_{\mathrm{EL}}}\right) \cdot I_{\mathrm{NSC,MA}}$$

o. Flow via Na^{+} transporter on the apical membrane of a marginal cell: $I_{\mathrm{Na,trans,MA}}$

$$I_{\mathrm{Na,trans,MA}} = -I_{\mathrm{Na,NSC,MA}}$$

References

1 Nin, F. *et al.* Computational model of a circulation current that controls electrochemical properties in the mammalian cochlea. *Proc Natl Acad Sci U S A* **109**, 9191-9196, doi:10.1073/pnas.1120067109 (2012).

2 Takeuchi, S., Ando, M. & Kakigi, A. Mechanism generating endocochlear potential: role played by intermediate cells in stria vascularis. *Biophys J* **79**, 2572-2582, doi:S0006-3495(00)76497-6 [pii] 10.1016/S0006-3495(00)76497-6 (2000).

3 Hibino, H. & Kurachi, Y. Molecular and physiological bases of the K^+ circulation in the mammalian inner ear. *Physiology (Bethesda)* **21**, 336-345, doi:10.1152/physiol.00023.2006 (2006).

4 Zdebik, A. A., Wangemann, P. & Jentsch, T. J. Potassium ion movement in the inner ear: insights from genetic disease and mouse models. *Physiology (Bethesda)* **24**, 307-316, doi:10.1152/physiol.00018.2009 (2009).

5 Konishi, T. & Mendelsohn, M. Effect of ouabain on cochlear potentials and endolymph composition in guinea pigs. *Acta Otolaryngol* **69**, 192-199 (1970).

6 Schulte, B. A. & Adams, J. C. Distribution of immunoreactive Na^+,K^+-ATPase in gerbil cochlea. *J Histochem Cytochem* **37**, 127-134 (1989).

7 Nakazawa, K., Spicer, S. S. & Schulte, B. A. Ultrastructural localization of Na,K-ATPase in the gerbil cochlea. *J Histochem Cytochem* **43**, 981-991 (1995).

8 Takeuchi, S. & Ando, M. Inwardly rectifying K^+ currents in intermediate cells in the cochlea of gerbils: a possible contribution to the endocochlear potential. *Neurosci Lett* **247**, 175-178 (1998).

9 Hibino, H. *et al.* Expression of an inwardly rectifying K^+ channel, Kir5.1, in specific types of fibrocytes in the cochlear lateral wall suggests its functional importance in the establishment of endocochlear potential. *Eur J Neurosci* **19**, 76-84, doi:10.1111/j.1460-9568.2004.03092.x (2004).

10 Estevez, R. *et al.* Barttin is a Cl^- channel beta-subunit crucial for renal Cl^- reabsorption and inner ear K^+ secretion. *Nature* **414**, 558-561, doi:10.1038/3510709935107099 [pii] (2001).

11 Rickheit, G. *et al.* Endocochlear potential depends on Cl^- channels: mechanism underlying deafness in Bartter syndrome IV. *EMBO J* **27**, 2907-2917, doi:emboj2008203 [pii] 10.1038/emboj.2008.203 (2008).

12 Crouch, J. J., Sakaguchi, N., Lytle, C. & Schulte, B. A. Immunohistochemical localization of

the Na-K-Cl co-transporter (NKCC1) in the gerbil inner ear. *J Histochem Cytochem* **45**, 773-778 (1997).

13 Sakagami, M. *et al.* Cellular localization of rat Isk protein in the stria vascularis by immunohistochemical observation. *Hear Res* **56**, 168-172 (1991).

14 Shen, Z. & Marcus, D. C. Divalent cations inhibit IsK/KvLQT1 channels in excised membrane patches of strial marginal cells. *Hear Res* **123**, 157-167, doi:S0378-5955(98)00110-5 [pii] (1998).

15 Davis, H. Some principles of sensory receptor action. *Physiol Rev* **41**, 391-416 (1961).

16 Hudspeth, A. J. How the ear's works work. *Nature* **341**, 397-404, doi:10.1038/341397a0 (1989).

17 Russell, I. J., Cody, A. R. & Richardson, G. P. The responses of inner and outer hair cells in the basal turn of the guinea-pig cochlea and in the mouse cochlea grown in vitro. *Hear Res* **22**, 199-216 (1986).

18 Kennedy, H. J., Evans, M. G., Crawford, A. C. & Fettiplace, R. Fast adaptation of mechanoelectrical transducer channels in mammalian cochlear hair cells. *Nat Neurosci* **6**, 832-836, doi:10.1038/nn1089 (2003).

19 Takeuchi, S. & Irimajiri, A. A novel, volume-correlated Cl^- conductance in marginal cells dissociated from the stria vascularis of gerbils. *J Membr Biol* **150**, 47-62 (1996).

20 Santi, P. A. & Lakhani, B. N. The effect of bumetanide on the stria vascularis: a stereological analysis of cell volume density. *Hear Res* **12**, 151-165 (1983).

21 Spicer, S. S. & Schulte, B. A. Novel structures in marginal and intermediate cells presumably relate to functions of apical versus basal strial strata. *Hear Res* **200**, 87-101, doi:10.1016/j.heares.2004.09.006 (2005).

22 Kelly, J. J., Forge, A. & Jagger, D. J. Contractility in type III cochlear fibrocytes is dependent on non-muscle myosin II and intercellular gap junctional coupling. *J Assoc Res Otolaryngol* **13**, 473-484, doi:10.1007/s10162-012-0322-7 (2012).

23 Takeuchi, S. & Ando, M. Voltage-dependent outward K^+ current in intermediate cell of stria vascularis of gerbil cochlea. *Am J Physiol* **277**, C91-99 (1999).

24 Ikeda, K. & Morizono, T. Electrochemical profile for calcium ions in the stria vascularis: cellular model of calcium transport mechanism. *Hear Res* **40**, 111-116 (1989).

25 Nin, F. *et al.* The endocochlear potential depends on two K^+ diffusion potentials and an electrical barrier in the stria vascularis of the inner ear. *Proc Natl Acad Sci U S A* **105**, 1751-1756, doi:10.1073/pnas.0711463105 (2008).

26 Adachi, N. *et al.* The mechanism underlying maintenance of the endocochlear potential by the K^+ transport system in fibrocytes of the inner ear. *J Physiol* **591**, 4459-4472, doi:10.1113/jphysiol.2013.258046 (2013).

27 Yoshida, T. *et al.* NKCCs in the fibrocytes of the spiral ligament are silent on the unidirectional K^+ transport that controls the electrochemical properties in the mammalian cochlea. *Pflugers Arch* **467**, 1577-1589, doi:10.1007/s00424-014-1597-9 (2015).

28 Yoshida, T. *et al.* The unique ion permeability profile of cochlear fibrocytes and its contribution to establishing their positive resting membrane potential. *Pflugers Arch* **468**, 1609-1619, doi:10.1007/s00424-016-1853-2 (2016).

29 Konishi, T. & Salt, A. N. Electrochemical profile for potassium ions across the cochlear hair cell membranes of normal and noise-exposed guinea pigs. *Hear Res* **11**, 219-233 (1983).

30 Wangemann, P. & Schacht, J. in *The Cochlea* (eds P. Dallos, A.N. Popper, & R. R. Fay) Ch. 3, 130-185 (Springer-Verlag, 1996).

31 Hibino, H. *et al.* An ATP-dependent inwardly rectifying potassium channel, KAB-2 (Kir4. 1), in cochlear stria vascularis of inner ear: its specific subcellular localization and correlation with the formation of endocochlear potential. *J Neurosci* **17**, 4711-4721 (1997).

32 Takeuchi, S., Ando, M., Kozakura, K., Saito, H. & Irimajiri, A. Ion channels in basolateral membrane of marginal cells dissociated from gerbil stria vascularis. *Hear Res* **83**, 89-100 (1995).

33 Takeuchi, S., Marcus, D. C. & Wangemann, P. Ca^{2+}-activated nonselective cation, maxi K^+ and Cl^- channels in apical membrane of marginal cells of stria vascularis. *Hear Res* **61**, 86-96 (1992).

34 Ashmore, J. F. & Meech, R. W. Ionic basis of membrane potential in outer hair cells of guinea pig cochlea. *Nature* **322**, 368-371, doi:10.1038/322368a0 (1986).

35 van Den Abbeele, T., Teulon, J. & Huy, P. T. Two types of voltage-dependent potassium channels in outer hair cells from the guinea pig cochlea. *Am J Physiol* **277**, C913-925 (1999).

36 Quraishi, I. H. & Raphael, R. M. Computational model of vectorial potassium transport by cochlear marginal cells and vestibular dark cells. *Am J Physiol Cell Physiol* **292**, C591-602, doi:10.1152/ajpcell.00560.2005 (2007).

37 Kakehata, S. & Santos-Sacchi, J. Membrane tension directly shifts voltage dependence of outer hair cell motility and associated gating charge. *Biophys J* **68**, 2190-2197, doi:10.1016/S0006-3495(95)80401-7 (1995).

38 Santi, P. A. & Muchow, D. C. Morphometry of the chinchilla organ of Corti and stria

vascularis. *J Histochem Cytochem* **27**, 1539-1542 (1979).
39 Schuknecht, H. *The inner ear.*, (Lea and Febiger, 1993).
